# Oral N-acetylcysteine decreases IFN-γ production and ameliorates ischemia-reperfusion injury in steatotic livers

**DOI:** 10.3389/fimmu.2022.898799

**Published:** 2022-09-05

**Authors:** Jedson R. Liggett, Jiman Kang, Suman Ranjit, Olga Rodriguez, Katrina Loh, Digvijay Patil, Yuki Cui, Anju Duttargi, Sang Nguyen, Britney He, Yichien Lee, Kesha Oza, Brett S. Frank, DongHyang Kwon, Heng-Hong Li, Bhaskar Kallakury, Andrew Libby, Moshe Levi, Simon C. Robson, Thomas M. Fishbein, Wanxing Cui, Chris Albanese, Khalid Khan, Alexander Kroemer

**Affiliations:** ^1^ MedStar Georgetown Transplant Institute, MedStar Georgetown University Hospital and the Center for Translational Transplant Medicine, Georgetown University Medical Center, Washington, DC, United States; ^2^ Department of Surgery, Naval Medical Center Portsmouth, Portsmouth, VA, United States; ^3^ Department of Biochemistry and Molecular & Cellular Biology, Georgetown University, Washington, DC, United States; ^4^ Microscopy & Imaging Shared Resource, Georgetown University, Washington, DC, United States; ^5^ Center for Translational Imaging, Georgetown University Medical Center, Washington, DC, United States; ^6^ Department of Oncology, Lombardi Comprehensive Cancer Center, Georgetown University Medical Center, Washington, DC, United States; ^7^ Department of Pathology, MedStar Georgetown University Hospital, Washington, DC, United States; ^8^ Division of Endocrinology, Metabolism, & Diabetes, University of Colorado Anschutz Medical Campus, Aurora, CO, United States; ^9^ Departments of Anesthesiology and Medicine, Beth Israel Deaconess Medical Center, Harvard Medical School, Boston, MA, United States; ^10^ Department of Radiology, MedStar Georgetown University Hospital, Washington, DC, United States

**Keywords:** NKT (natural killer T) cell, hepatic steatosis, IFN-gamma, ischemia-reperfusion injury (IRI), N-acetylcysteine (NAC), phasor-FLIM, gadoxetate disodium, liver transplantation

## Abstract

Type 1 Natural Killer T-cells (NKT1 cells) play a critical role in mediating hepatic ischemia-reperfusion injury (IRI). Although hepatic steatosis is a major risk factor for preservation type injury, how NKT cells impact this is understudied. Given NKT1 cell activation by phospholipid ligands recognized presented by CD1d, we hypothesized that NKT1 cells are key modulators of hepatic IRI because of the increased frequency of activating ligands in the setting of hepatic steatosis. We first demonstrate that IRI is exacerbated by a high-fat diet (HFD) in experimental murine models of warm partial ischemia. This is evident in the evaluation of ALT levels and Phasor-Fluorescence Lifetime (Phasor-FLIM) Imaging for glycolytic stress. Polychromatic flow cytometry identified pronounced increases in CD45+CD3+NK1.1+NKT1 cells in HFD fed mice when compared to mice fed a normal diet (ND). This observation is further extended to IRI, measuring *ex vivo* cytokine expression in the HFD and ND. Much higher interferon-gamma (IFN-γ) expression is noted in the HFD mice after IRI. We further tested our hypothesis by performing a lipidomic analysis of hepatic tissue and compared this to Phasor-FLIM imaging using “long lifetime species”, a byproduct of lipid oxidation. There are higher levels of triacylglycerols and phospholipids in HFD mice. Since N-acetylcysteine (NAC) is able to limit hepatic steatosis, we tested how oral NAC supplementation in HFD mice impacted IRI. Interestingly, oral NAC supplementation in HFD mice results in improved hepatic enhancement using contrast-enhanced magnetic resonance imaging (MRI) compared to HFD control mice and normalization of glycolysis demonstrated by Phasor-FLIM imaging. This correlated with improved biochemical serum levels and a decrease in IFN-γ expression at a tissue level and from CD45+CD3+CD1d+ cells. Lipidomic evaluation of tissue in the HFD+NAC mice demonstrated a drastic decrease in triacylglycerol, suggesting downregulation of the PPAR-γ pathway.

## Introduction

Hepatic ischemia-reperfusion injury (IRI) remains a significant complication in both surgical liver resection and liver transplantation. IRI is a complex and incompletely understood process that leads to the disruption of cellular integrity with the release of damages-associated molecular patterns and subsequent innate immune system activation (1), though identification of key mediators remains the point of ongoing research. In liver transplantation, the degree of IRI is more profound in the setting of hepatic steatosis and has historically been shown to increase the risk of primary non-function, dysfunction, and reduce graft and patient survival (1). Unfortunately, due to the increasing demand for liver transplantation and a lack of transplantable livers, “marginal” allografts, including those with hepatic steatosis, are being considered despite an elevated risk of IRI.

Currently, there are few available therapeutic options in the prevention and treatment of IRI, and N-acetylcysteine (NAC), a thiol-containing synthetic compound, remains a mainstay in the treatment. While the efficacy of NAC has been established in the treatment of acetaminophen toxicity and has shown some efficacy against IRI and fulminant liver failure, its mechanisms, optimization of treatment, and impact on long-term survival remain understudied (2). Given the limited treatment options for IRI and the increasing demand for liver transplantation, significant research efforts are required to not only identify key mediators and therapeutic options for IRI, but also accurately evaluate donor allografts for high-risk stigmata in order to safely utilize these “marginal” organs while decreasing the associated risk.

While no standard exists, donor allograft biopsy is frequently performed to aid in allograft selection, and greater than 30% hepatic macro steatosis is often a deterrent for allograft use. However, a recent study by Patel et al. has shown that steatotic allografts can be successfully utilized with appropriate recipient selection (3). To aid in the selection process, advanced methods to investigate the integrity and function of the allograft may be essential for the use of marginal allografts. Both Magnetic Resonance Imaging (MRI) and Phasor Fluorescence Lifetime Imaging (FLIM) are promising modalities to evaluate liver function and injury. T2 mapping by MRI has previously been shown as a feasible, non-invasive technique in assessing acute liver injury (4), while FLIM has been demonstrated to assess for liver injury more accurately *via* the evaluation of steatosis, fibrosis (5), and glycolytic activity (6) in experimental models.

Invariant natural killer T (NKT) cells are a unique group of cells that can recognize both self and lipid antigens that are presented to them by MHC class I-like CD1d molecules (7). Type I invariant Natural Killer T-cells (NKT1 cells) not only express CD1d, but also express markers of both T-cells (CD3+) and conventional NK cells (NK1.1+) and can potently secrete IFN-γ and TNF-α (8–10). NKT1 cells have emerged as key mediators in hepatic IRI (10, 11) and have been shown to worsen this process in a NAFLD murine model, despite lower baseline populations (1). Interestingly, it is also known that NKT1 cells express a wide variety of activating antigens including endogenous lipid antigens such as phosphatidylcholine (12–15), which may lead to an enhanced response to IRI. Thus, preventing activation of NKT1 cells following IRI in NAFLD may aid in developing new therapeutic approaches that merit further investigation. In addition to the known benefits in IRI, NAC has also been demonstrated to alter metabolism and have benefits in the treatment of metabolic syndrome, in addition to the known benefit in IRI. However, the immunomodulatory capacity of NAC in liver injury remains understudied.

Given the activation of NKT1 cells by lipid antigens and potential roles of NKT1 cells in hepatic IRI, we hypothesized that NAC supplementation would ameliorate IRI in a high-fat diet (HFD) murine model *via* decreased activation of NKT1 cells. In our present work, we utilize advanced Phasor-FLIM imaging to interrogate tissue following IRI and further evaluate the innate immune response to IRI. This study successfully identifies NKT1 cells and IFN-γ as mediators in IRI in the setting of hepatic steatosis and shows an improved clinical phenotype following NAC supplementation at least in part secondary to reduced NKT1 cell activation.

## Materials and methods

### Diets

The normal diet (ND), #5001, was purchased from Lab Diet, St. Louis MO, (4.09 kcal/gram,13.4% kJ/fat). The high-fat diet (HFD), 58Y1 blue, was purchased from TestDiet, St. Louis, MO (5.10 kcal/gram, 60% kJ/fat). HFD was stored in refrigerated conditions at 4°C. NAC was purchased from Sigma, St. Louis, MO and was added to the treatment group drinking water in a concentration of 10 gram/liter.

### Animals

All animal procedures in this study were fully approved by the Georgetown University Institutional Animal Care and Use Committee under protocol number 2016-1351. C57BL/6 mice were obtained at five to six weeks of age from Charles River Laboratories and The Jackson Laboratory, Bar Harbor, ME. The mice were maintained in the Division of Comparative Medicine at Georgetown University Medical Center, with a standard 12-hour light-dark cycle. Male mice were used for all experiments. Food and water were provided *ab libitum* in the four experimental groups: ND, ND + NAC, HFD, and HFD + NAC. The ND diets were administered at the beginning of the study and maintained for 19-23 weeks. The HFD was started at six to seven weeks of age and continued for 19-23 weeks. NAC drinking water was administered to the ND + NAC and HFD + NAC groups following three weeks of ND or HFD and maintained for 16-20 weeks. The NAC-supplemented drinking water was changed weekly. Consumption of the HFD and individual animal weights were measured weekly. Additional C57BL/6 mice were purchased at 25 weeks of age from The Jackson Laboratory, Bar Harbor, ME on a ND, #5K52 from Lab Diet (3.50 kcal/gram, 16.6% fat), for supplemental use in select groups.

### Partial warm IRI model

All procedures performed on C57BL/6 mice were fully approved by the Georgetown University Institutional Animal Care and Use Committee under protocol number 2016-1351. Mice were anesthetized with 2% isoflurane and oxygen inhalation. A midline laparotomy was performed, and an atraumatic micro clip was placed across the hepatic hilus, which interrupted the blood supply to the left and median lobes of the liver. The abdomen was temporarily closed with skin staples, and the mice remained anesthetized. A 45-minute ischemic time was utilized to represent a clinically relevant model, more closely corresponding to the average warm ischemia time in human liver transplantation. This also prevented excess animal death under anesthesia, given the metabolic changes associated within the HFD model. Following 45 minutes of partial hepatic ischemia, the clip was removed to initiate hepatic reperfusion. The abdominal wall was closed with sutures, the skin was reapproximated with staples, and the animals were returned to their cage. All mice undergoing Magnetic Resonance Imaging following IRI, had their abdominal wall and skin reapproximated with sutures. After 24 hours of reperfusion, mice were anesthetized and underwent a second laparotomy. Whole blood was obtained *via* direct cardiac puncture as a terminal procedure. The left lobe of the liver and the whole spleen were collected. Sham controls underwent the same procedure but without vascular occlusion.

### Magnetic resonance imaging

Magnetic resonance imaging (MRI) was performed in the Georgetown-Lombardi Preclinical Imaging Research Laboratory on either a 7T/20 Avance III/ParaVision 5 or a 7T/30 USR Avance NEO/ParaVision 360 (S10 OD025153) scanner. The mice were anesthetized (1.5% isoflurane in a gas mixture of 30% oxygen and 70% nitrous oxide) and placed on a custom-manufactured (ASI Instruments, Warren, MI) stereotaxic device, with built-in temperature and cardio-respiratory monitoring as described (B, C), and compatible with a 40 mm Bruker mouse body volume coil.

*Adipose tissue.* MRI of abdominal and liver fat was performed with a three-dimensional rapid acquisition with rapid enhancement (3D-RARE) sequence in the coronal orientation with the following parameters: TR: 2855 ms, TE: 12 ms, RARE Factor: 4, Matrix: 220 x 220, FOV: 50 mm x 40 mm, Averages: 4, Slice thickness: 0.75 mm, Slices: 50 and respiratory gating Quantification of visceral fat depots in the imaging datasets was performed by thresholding and voxel-counting with ImageJ software (NIH), as previously described (16–18). We used a maximum intensity projection algorithm of the 3D-reconstructed image with an intensity threshold intended to segment fat only. The abdominal region analyzed was defined by superior and inferior anatomical landmarks, that is, the proximal border of the left kidney and the convergence of the left and right common iliac veins, respectively. The lateral landmark was the abdominal wall, avoiding subcutaneous fat. The percentage area corresponding to fat depots within the abdomen was calculated *via* the sum of the visceral fat voxels versus total abdominal voxels. Mouse liver fat was measured by quantifying the mean intensity of the region of interest (ROI) localized on the mouse liver placed in homogenous areas, avoiding structures such as large vessels and ducts. ROIs on three separate slices were selected and averaged for each mouse. For imaging of the nuchal fat, a 3-D T1-weighted RARE sequence in the sagittal orientation was run with TR: 2437 ms, TE: 15 ms, FA: 74.1, Matrix: 256 x 256, FOV: 40 mm x 40 mm, Slice thickness: 0.5 mm, Averages: 4, Slices: 35. The mean intensity of nuchal brown adipose tissue (BAT) and white adipose tissue (WAT) was measured by localizing ROIs on the BAT and the WAT. ROIs on three separate slices were selected and averaged for each mouse.

*Dynamic Contrast-Enhanced MRI (DCE-MRI) using Gadoxetate Disodium (Eovist^®^, Bayer)*. DCE-MRI was performed with a T1-weighted RARE protocol in the coronal orientation, without respiratory gating, with TE: 8.2 ms, TR: 400 ms, slice thickness: 1 mm, matrix: 128 x128, FOV: 40 mm x 40 mm, rare factor: 2, fat suppression and a duration of 25 s. Ten baseline (pre-contrast) scans were run, followed by subcutaneous parenteral administration of 0.025 mmol/kg Eovist. Immediately after injection, 60 MRI scans were acquired repetitively over approximately 30 minutes, and the uptake and excretion of contrast was measured over time. Relative liver enhancement (RLE) was used to quantify hepatic function. Briefly, the mean intensity of regions of interest (ROI) localized in homogenous areas of the liver were quantified in baseline and post-injection images. Liver function was calculated using the formula *RLE = (SI_Liver enh_ – SI_Liver unenh_)/SI_Liver unenh_ x 100.* The area-under-the-curve (AUC) and the slope were derived from the corresponding RLE data. The slopes were measured for the first 20 minutes post-injection, where after this time point, the curves reached their plateau. Both the AUC and the slopes were calculated with a 95% confidence interval.

*T2 mapping* A two-dimensional multi-echo multi-slice sequence in the axial orientation was used for T2 mapping with TR: 2000 ms, Flip Angle: 180 deg, Averages: 2, and the following echo times, TE: 10, 20, 30, 40, 50, 60, 70 and 80 ms, with respiratory gating. In order to measure T2 relaxation times, we used the Paravision360 Image Sequence Analysis tool to generate T2-fit and T2-maps. ROI were localized on the left and right liver areas in four slices per mouse, excluding non-homogenous structures such as large hepatic vessels or ducts, after which T2 values were quantified and averaged.

### Measurement of alanine aminotransferase (ALT)

Whole blood was obtained by direct cardiac puncture as a terminal procedure. ALT levels were measured using a multichannel analyzer, Alfa Wassermann Vet Axcel, from the clinical diagnostics laboratory of VRL Maryland, LLC.

### Histology and immunohistochemistry

Hematoxylin and eosin (H&E) and immunohistochemical staining was performed on five-micron sections from formalin-fixed paraffin-embedded liver tissues. H&E staining was completed, and samples were then rehydration through a graded alcohol series using the Autostainer XL (Leica Biosystems). Gr-1 and CD68 staining was performed using the ImmPRESS Goat anti-rat Polymer Detection Kit (peroxidase) (Vector laboratories, MP-7444) and horseradish peroxidase-labeled polymer from (Dako, K4003) for Gr-1 and CD68 respectively. Gr-1and CD68 cell counts were performed manually in five high-powered field sections of 20x magnification using an Olympus BX41 light microscope. Cell counting was performed in a blinded manner.

### Fluorescence lifetime imaging and second harmonic generation imaging

*Instrumentation*. The autofluorescence lifetime images of the liver sections (5 µm thick) were measured using a modified Olympus FVMPERS (Waltham, MA) microscope equipped with a Spectra-Physics Insight X3 (Milpitas, CA) laser and FastFLIM (ISS, Champaign, IL) acquisition card. The samples were excited with the 740 nm laser line using a 20X air objective (LUCPLFLN 0.45NA, Olympus) applying a two-photon excitation scheme. The fluorescence was collected using the DIVER (Deep Imaging *Via* Enhanced Recovery) detector (19–21) assembly and recorded using a FastFLIM card (ISS, Champaign, IL). The pixel dwell time for the acquisitions was 20 µs, and the images were taken with sizes of 256x256 pixels with a field of view of 318.8 µm (Zoom =2X). To obtain a high signal-to-noise ratio, 16 frames were collected for each area. The data from each pixel were recorded and analyzed using the SimFCS software (Laboratory for Fluorescence Dynamics, University of California, Irvine, CA). The FLIM data were collected using the passive mode, where the raster scanning was done using the Olympus software, and the images were collected using the FLIMBox/FastFLIM system, and the scanning parameters were matched to ensure proper image acquisition. SHG and FLIM images were obtained using two separate filter assemblies in DIVER and further separated based on their lifetime and phasor position.

*Phasor Fluorescence Lifetime Imaging (FLIM) Methodology.* The Phasor approach to FLIM is a fit free method of analysis where fluorescence decay information collected at each pixel of an image is transformed to the phasor plot and designated a coordinate based on the lifetime (22–25). Being a fit free method, phasor is a much faster analysis method and is used increasingly in biological fluorescence imaging with a large data set. The phasor transformation results in the formation of a heat map in the phasor plot where each pixel of an image is represented by a single phasor point and segmentation and filtering methods and of image analysis can be applied without loss of image definition of the original intensity images. Recent advances in analysis techniques allow us to quantify the contribution of multiple components and this is applied in this work (26–29).

A brief description of the method follows below. A much more detailed description of the method, along with individual analysis schemes, flowcharts, and assumptions for both phasor-FLIM quantification and SHG analysis is explained in the supplemental information. FastFLIM (ISS, Champaigne, IL) is a frequency domain FLIM imaging instrument. In frequency-domain fluorescence lifetime measurements, the transformation to the phasor plot uses the following relations,


3 $$g_{i,j}\left(\omega \right)=m_{i,j}\cdot cos\left(\phi_{i,j}\right)$$

4 $$s_{i,j}\left(\omega \right)=m_{i,j}\cdot sin\left(\phi_{i,j}\right)$$

where, *g_i,j_(ω)* and *s_i,j_(ω)* are the X and Y coordinates of the phasor plot, respectively, and *m_i,j_* and *ϕ_i,j_*are the modulation and phase of fluorescence signal at the image pixel *i, j*. The longer lifetime is represented by the larger phase angle in the Phasor plot – movement towards S=0, G=0 around universal semi-circle. The distribution of phasor points originating from FLIM measurements appears on (for mono-exponential decays) or inside (for multi-exponential decays) the universal circle (please refer to SM1 in the phasor supplemental methods). The linear combinations are shown by the blue. If each component has a multi-exponential decay, its location will be represented by phasors not in the universal circle, but the principle of linear combination remains valid. More details can be found in the phasor section of the supplemental methods.

According to this principle, if a pixel of an image has multiple components originating from multiples species, then the position of the corresponding phasor point is inside the polygon whose vertices are occupied by the Phasor points originating from the individual components. The distance of the image phasor point from any of the vertices is reciprocal to the fractional intensity component of that particular component. The higher the fractional intensity contribution of that particular species towards the total intensity of the image pixel, the closer the phasor point of that image pixel is to the corresponding component phasor position (24, 30, 31). For a three component system, an algebraic solution of this system exists, and this allows the breakdown of a Phasor cloud from an image to the individual fractional intensity components (28). Our recent work created a framework where the phasor clouds originating from multiple components can be quantitatively resolved, and their individual fractional intensities can be calculated (26, 28, 29). For a system where the quantum yield can be assumed based on their lifetimes or can be quantified – the fractional intensity ratios can be modified to molar fraction ratios. Please see supplemental methods for additional details of the analysis and stepwise processes.

In this work, the positions of the three original phasor positions were selected based on previously published work. Free and protein bound NADH have a lifetime of 0.4 ns and ~3.4 ns, respectively, and their positions are on universal semicircle (32), and are shown by the blue and green circles, respectively (**Figure 3A**). The line joining the two cursors is named metabolic trajectory (33). The long lifetime species (LLS) has a lifetime of ~8ns, and the corresponding phasor position is selected by the red circle (26, 27, 33, 34). In the presence of LLS, the autofluorescence FLIM phasor in the NADH channel shifts away from the metabolic trajectory and inside the triangle whose vertices are occupied by the central positions of red, green, and blue circles. As mentioned above and in supplemental methods (Phasor section) – we have used multiple component analysis to obtain the quantitative information about the concentration ratio of free and protein-bound NADH and fractional intensity of NADH.

### Targeted lipidomic profiling

Snap-frozen liver tissue was dissolved in 300 µL of extraction buffer (IPA) and subjected to two cycles of freezing and rapid thawing into a 37°C water bath or 90 seconds. Then, tissue samples were sonicated at 30 kHz for 30 seconds and mixed with 100μl of ice-chilled isopropyl alcohol (IPA) with internal standards (IS). Upon 30 minutes of incubation on ice and additional incubation at −20°C for 30 minutes, the tissue in suspension was spun down at 14000xg at 4°C for 20 minutes to collect the supernatant. Targeted LCMS-MS was performed using Xbridge Amide 3.5 μm, 4.6 × 100 mm column (Phenomenex) online with a triple quadrupole mass spectrometer (5500 QTRAP, SCIEX) equipped in the multiple reaction monitoring (MRM) modes. Each metabolite, declustering potential, collision energies, cell exit potential, and entrance potential were optimized to acquire maximum ion intensity using Analyst 1.6.3 software (SCIEX, United States).

To ensure the most intense precursor and fragment ion pair selection, for the MRM-based quantitation, we ranked individual analyte signal intensities for all MRM Q1/Q3 ion pairs of that specific analyte. To obtain metabolite ratio, we used normalized peak area of endogenous metabolites within samples with normalized IS for every class of lipid molecule. Appropriate measures were taken to randomize sample queue and to assess sample carryover. Pooled quality control (pooled QC) samples were injected periodically to check for instrumental variation and National Institute of Standards (NIST) plasma samples were injected for lipidomic data analysis. Data normalized to QC variance. MultiQuant 3.0.3 (Sciex) software was used to obtain QC normalized data and imputed MRM data. To determine relative quantification values of analytes, the ratio of peak areas of sample transitions normalized to the peak area of the IS specific for every class was computed. Data post-processing statistical analysis including heatmap, volcano plot, and ANOVA was conducted with the software MetaboAnalyst (35). Product numbers for all reagents are listed in **Supplemental Table 1**.

### Cell preparation and polychromatic flow cytometry

The left lobe of the liver was collected into RPMI-1640 culture medium (Gibco). Liver tissues were harvested, passed through a 70-μm cell strainer (Fisher Scientific), and leukocyte fractions were isolated *via* Percoll (Cytiva) density gradient. Samples were centrifuged at 1000g (25°C), without brake for 20 minutes, and the upper layer was carefully discarded. The leukocyte layer was washed and resuspended in 1x PBS (Gibco). Liver leukocytes were stained with the following antibodies (BioLegend, San Diego, CA) for flow cytometry analysis: Alexa Fluor700 conjugated CD45, Brilliant Violet 510 conjugated CD 4, Brilliant Violet 421 conjugated CD8, Brilliant Violet 605 conjugated NK1.1, PE-CD1d-tetramer (NIH Core Tetramer Facility), and Fluorescein isothiocyanate-conjugated CD3. Data were acquired using a BD FACSAria III Cytometer (BD Biosciences) at our Flow Cytometry & Cell Sorting Shared Resource. Any samples with viability of 60% or lower (as determined by staining with live dead marker Zombie NIR™, BioLegend) were excluded from all analyses. FlowJo v10 (BD, Franklin Lakes, NJ) was used for all subsequent data analyses. Product numbers for all reagents are listed in **Supplemental Table 1**.

### Cytokine stimulation and intracellular cytokine staining

Intracellular staining for the detection of cytokines was carried out from liver leukocytes. Approximately 1x10^6^cells/ml RPMI supplemented with 10% FBS, 1% Penicillin Streptomycin, and 0.5% Gentamycin were cultured for 20 hours at 37°C in a cell culture flask. Recombinant mouse cytokine concentrations used were 10 ng/ml IL-12, 50 ng/ml IL-18, and 50 ng/ml IL-15 (R&D Systems). Cells were stimulated for 4 hours with Cell Activation Cocktail containing phorbol-12-myristate-13-acetate (PMA, 50 ng/ml) and ionomycin, (BioLegend) in the presence of 5 μg/ml brefeldin A (BioLegend). Following stimulation, the cells were stained with the following antibodies to detect cytokines: Brilliant Violet 650™-conjugated anti-IFN-γ (BD Biosciences) and Brilliant Violet 605™-conjugated anti-TNF-α (BioLegend). Cells were fixed (IC Fixation buffer, Invitrogen) and permeabilized (Permeabilization buffer, Invitrogen) according to the manufacturer’s instructions.

### Real-time polymerase chain reaction

To assess the effect of IRI on mouse-specific cytokines and chemokines, we used the SYBR-green based RT² Profiler™ Polymerase Chain Reaction (PCR) Array Mouse Cytokines & Chemokines (PAMM-150ZC-12, Qiagen) to evaluate the gene expression of 84 different cytokines and chemokines. For this experiment, mice liver specimens collected 24 hours-post sham and IRI surgeries and stored in the Allprotect Tissue Reagent (Qiagen) were used for the total RNA extraction. All tissues were lysed using 1.0 mm Zirconia/Silica beads (BioSpec Products) in FastPrep 24 Tissue Homogenizer (MP Biomedical). We extracted total mouse liver RNA as instructed in the RNeasy Mini kit with RNase-free DNase set (Qiagen). RNA quality and quantity were assessed using 2100 Agilent Bioanalyzer, and RNA samples with RNA integrity number (RIN) ≥ 7 were used for the PCR arrays.

For each RT² Profiler™ PCR Array, 1.25 µg of total RNA was pooled from at least three separate mice livers in an equal proportion. We performed standard procedures to eliminate mouse genomic DNA, synthesize first-strand and set up RT^2^ profiler PCR array for cytokines and chemokines. Raw Ct values from the array were analyzed using the GeneGlobe web tool (https://geneglobe.qiagen.com/us/) to check the quality of the PCR arrays, normalize threshold cycle (Ct) values using Beta-2-Microglobulin (B2m) as a housekeeping gene and estimate fold regulation with ND (Sham) as a control group. Unsupervised hierarchical clustering for the 2^-ΔΔCt^ was performed using ClustVis (36) to identify gene clusters specific to sham and IRI groups under two different diet regimens. Venn diagrams for genes with fold upregulation ≥ 2 were created using Venny (37) to identify common genes among all the IRI groups and exclusively diet-specific gene signatures. Product numbers for all reagents are listed in **Supplemental Table 1**.

### Statistical analysis

All data are expressed as mean ± standard error of the mean (SEM). Comparison between groups was performed by a Mann-Whitney U test unless otherwise mentioned in the figure legend. Statistical significance was established at p<0.05 with all p-values being two-sided and stratified in the following order: “*” 0.05 to 0.01, “**”0.01 to 0.001, “***” 0.001 to 0.0001, and “****” <0.0001. Prism Software (GraphPad, Inc. San Diego) was used for all statistical analyses.

## Results

### NAC supplementation clinically reduced hepatic steatosis and facilitated Eovist^®^ uptake during hepatic MRI

To establish the efficacy of our HFD murine model, we first examined trends in body weight and food consumption within our model. HFD mice gained significantly more weight than ND mice and this effect was significantly reduced with oral NAC supplementation (**Supplemental Figure 1A).** There were no statistically significant differences in food consumption between HFD and HFD + NAC, although HFD + NAC mice had a trend of higher food consumption. The average NAC water consumption was 139 mL/week/cage of five mice in the HFD + NAC mice and there was no significant difference as compared to ND + NAC water consumption. We further assessed blood glucose measurements; HFD mice had significantly higher blood glucose measurements starting at twelve weeks of age compared to HFD + NAC mice (p<0.05) (**Supplemental Figure 1B**).

We next assessed the adiposity of the liver, the abdomen, and the nuchal areas of mice from each group, *in vivo*, using 3D-RARE MRI. HFD mice presented with a significant accumulation of hepatic steatosis and visceral abdominal fat and accumulation of white adipose tissue (WAT) in the nuchal region. NAC treatment prevented the development of hepatic steatosis and supported the transformation of WAT into brown adipose tissue (BAT) (**Supplemental Figure 2**). We then correlated these findings with H&E-stained liver sections evaluated by an expert pathologist. Consistently, hepatic steatosis varied between both ND and ND + NAC when compared to HFD mice, with HFD mice demonstrating 80% hepatic steatosis on average (p<0.0001). This difference was significantly reduced in mice on NAC supplementation, which resulted in a 50% reduction in hepatic steatosis (**Supplemental Figure 3**).

**Figure 1:**
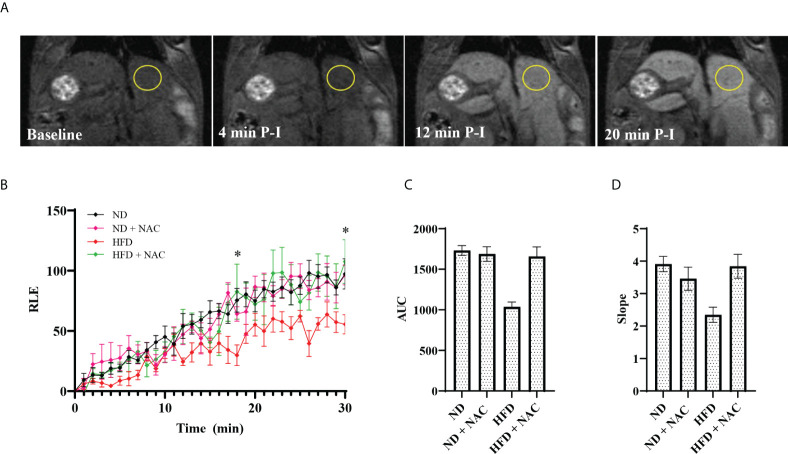
Effects of HFD on liver function as assessed by DCE-MRI with the contrast agent Gadoxetate Disodium (Eovist^®^ Bayer). **(A)** Representative images of 26-30 week old male mice undergoing DCE-MRI, showing T1-weighted images performed before Eovist^®^ injection and at 4, 12 and 20 minutes post-injection (P-I). The yellow circle denotes a typical region of interest used to measure mean intensity. **(B)** Time course of relative liver enhanced T1 signal intensities after Eovist^®^ injection, comparing mice on ND (n=4), ND + NAC (n=5), HFD (n=5), and HFD + NAC (n=5). The differences in mean intensity between HFD and all the other groups (ND, ND + NAC and, HFD + NAC) were significant at 18- and 30-minutes post-injection. **(C)** Area under the curve (AUC) calculated from the data in **(B)**. **(D)** The slope of T1 signal intensity increase for each group shown, corresponding to the rates of change within the first 20 min after Eovist^®^ injection. Statistical significance was established using a one-way ANOVA multiple comparisons test. “*” 0.05 to 0.01.

Given the profound reduction of hepatic steatosis in the HFD + NAC mice, we next assessed the baseline liver function of our HFD murine model by performing DCE-MRI with Eovist^®^. HFD fed mice showed a significant decrease in relative liver contrast enhancement as compared to ND mice (**Figure 1**). These data, expressed as AUC analyses, correlated strongly with other imaging parameters such as *in vivo* and *ex vivo* liver fat content. Corresponding contrast enhancement slopes were calculated during the first 20 minutes post-injection, which represents the transitional phase before the contrast levels begin to plateau during the hepatobiliary phase. These data were strongly correlated with the AUC and overall hepatic relative liver enhancement values, where the rates of T1 enhancement in the HFD group were slower and failed to achieve the signal intensity levels observed in the ND mice. However, the HFD + NAC mice exhibited contrast uptake comparable to that observed in ND mice. It is important to note, that the differences in mean intensity between HFD and all the other groups (ND, ND + NAC and, HFD + NAC) were significant at 18- and 30-minutes post-injection. Collectively, these data indicate the deficits in Eovist uptake in the HFD mice are restored in HFD + NAC mice to a level consistent with ND mice.

### Hepatic ischemia-reperfusion injury in a HFD model is attenuated following oral NAC supplementation

**Figure 2:**
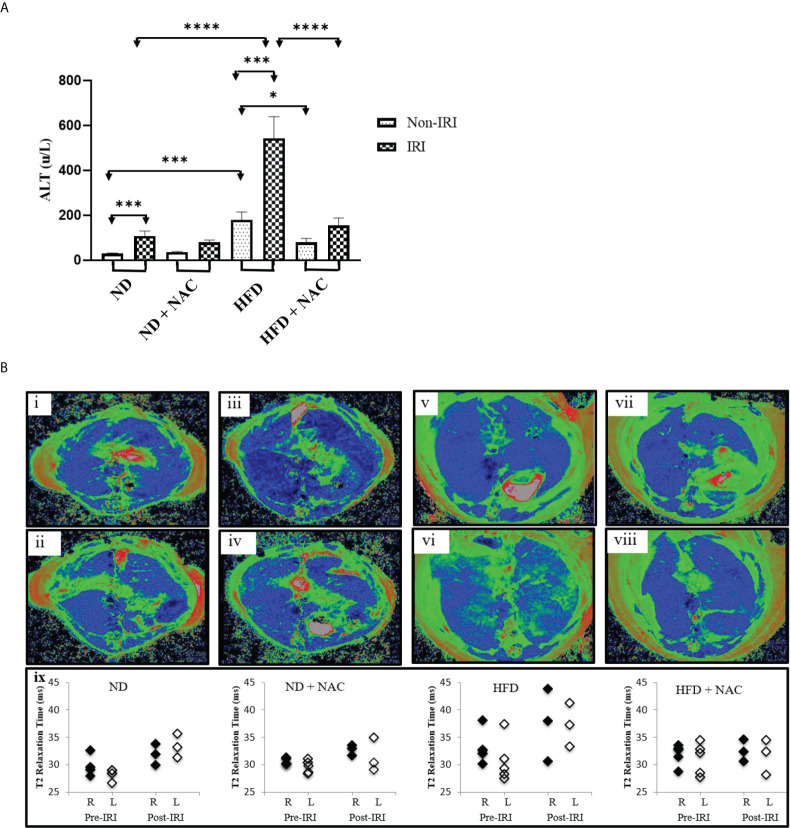
HFD Mice are prone to more severe IRI, which is significantly improved with NAC supplementation. **(A)** Serum ALT level following 24 hours of reperfusion. Number of mice per group is as follows: ND=11, ND (IRI)=13, ND + NAC= 5, ND + NAC (IRI)=10, HFD=5, HFD (IRI)=15, HFD + NAC, HFD + NAC (IRI)=8. **(B)** Representative images of baseline T2 mapping of mice on i) ND, iii), ND + NAC, v) HFD and vii) HFD + NAC. Representative images of T2 mapping twenty-four hours after IRI surgery of ii) ND, iv) ND + NAC, vi) HFD and viii) HFD + NAC. Blue represents liver parenchyma while green denotes edema. Graphical representation (xi) of the average of the T2 relaxation times of ROIs placed on the right and left areas of the corresponding livers (n=3-5 per group). “*” 0.05 to 0.01, “***” 0.001 to 0.0001, and “****” <0.0001.

Partial warm IRI was induced in mice from all groups. Both ND and HFD groups experienced a significant elevation in serum ALT levels as compared to sham-operated mice (p<0.0001 and p=0.0009, respectively). However, HFD mice experienced a significantly sharper rise in serum ALT following IRI than did ND mice (79 u/L *vs*. 408 u/L, p<0.0001), suggesting a higher degree of injury. We then evaluated the role of NAC supplementation in the prevention of IRI and demonstrated a significant reduction in serum ALT following IRI in HFD + NAC mice as compared to HFD mice alone (201.5 u/L *vs.* 408 u/L, p<0.0001), and in fact, there was no significant difference between ND IRI and HFD + NAC IRI groups, suggesting a normalization of injury (**Figure 2A**).

Representative mice from each group were subjected to T2-weighted MRI prior to the terminal procedure to assess the magnitude, distribution, and bilaterality of hepatic edema and associated inflammation, and pre-and post-IRI T2 maps were generated. Specifically, baseline T2 mapping of the liver was performed on ND and HFD, with and without NAC. Twenty-four hours after partial hepatic IRI surgery, the mice again underwent T2 mapping. The T2 maps were generated, and the T2 values quantified. Pre-IRI T2 values were similar between ND mice and ND + NAC mice, while the T2 values trended towards a slight increase in the livers of mice on HFD (**Figure 2B**). Ischemia-reperfusion injury exposure did not affect the T2 values of either the ND or ND + NAC livers. Conversely, the hepatic T2 values of mice on HFD were noticeably elevated after IRI, while the T2 values of HFD + NAC were comparable to those observed with ND, both before and after IRI. It is important to note that baseline and post-IRI T2 values were not significantly different between the right and left liver lobes in any group. Overall, the T2 maps enabled the quantification and visualization of hepatic edema and inflammation resulting from IRI in HFD mice, which demonstrated an increase in hepatic T2 relaxation times in HFD as compared to ND mice. NAC ameliorated the impact of IRI in HFD mice, making T2 values comparable to those seen in the ND + NAC mice.

We further evaluated the liver tissue using immunohistochemistry to evaluate for neutrophil (Gr-1+) and macrophage (CD68+) influx in mice treated with NAC and underwent IRI. We have previously demonstrated that there is a significant increase in Gr-1 and CD68 positive cells in HFD IRI mice compared to ND IRI mice (38). We further established that HFD + NAC IRI mice have an average of a 45% reduction in Gr-1+ cell counts (p<0.0001) and an average of a 35% reduction in CD68 cells following IRI (p<0.0001) (data not shown). Representative immunohistochemical stained images are shown in **Supplemental Figure 3C**.

### Phasor-FLIM imaging reveals marked differences in glycolysis, successfully corresponding to the degree of hepatic IRI

**Figure 3:**
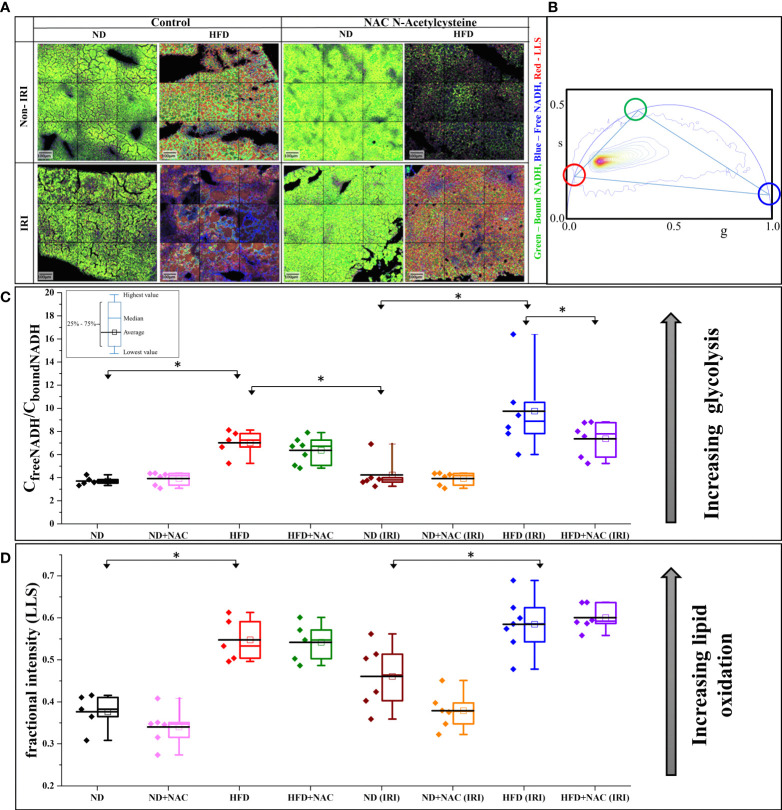
IRI results in a higher degree of glycolytic stress in HFD mice. **(A)** Color mapped Phasor FLIM images of the ND and HFD liver samples in the absence (left) and presence (right) of NAC supplementation. The top row shows the sham samples, and the bottom row shows the IRI samples. More green, blue and red color represents more protein-bound NADH, free NADH and long lifetime species (LLS), respectively. **(B)** Phasor plot of autofluorescence FLIM in liver samples – the three colored circles represent the lifetime position of the three components: free and bound NADH, and LLS. **(C)** The concentration ratio of free to bound NADH was calculated from each mouse. The higher value represents more glycolytic stress. There is a higher level of glycolytic stress in HFD and HFD (IRI) samples. This is reduced in the HFD + NAC (IRI) samples, but not the HFD + NAC samples, respective to their HFD counterparts. **(D)** The fractional intensity of LLS calculated using three-component calculations to show the increasing LLS in high fat liver and how that increases with IRI (n=5-7 mice per group for both analyses). “*” 0.05 to 0.01.

Given recent advances in Phasor FLIM and SHG imaging of steatotic human and mouse liver tissue (39), we sought to employ this methodology to evaluate the differences in liver tissue following IRI as a novel approach to evaluate liver injury. Samples were examined for glycolysis and lipid oxidation based on the Phasor-FLIM signatures. A higher ratio of free to protein-bound NADH is indicative of increased glycolysis (25, 40–43) and a larger amount of long lifetime species (LLS) is indicative of higher lipid peroxidation (33, 34). The FLIM images (**Figure 3A**) were colored according to the three cursor positions for bound NADH (green), free NADH (blue), and the LLS (red) selected using the phasor map (**Figure 3B**).

We first evaluated the models for increased glycolysis, as demonstrated by free NADH (blue). Visually, there is increasing blue color present in all groups following IRI, while the ND, ND + NAC and HFD + NAC groups show more green color, thus more bound NADH. These data, when converted to the concentration ratio of free/bound NADH (**Figure 3C**), establish that the ratio increases in HFD mice compared to ND mice and this is further exacerbated following IRI, again demonstrating a greater ratio of free/bound NADH ratio in HFD IRI mice compared to ND IRI mice. This increasing ratio correlates to higher glycolytic stress, thus a greater degree of IRI. Importantly, glycolytic stress is significantly reduced in the HFD + NAC IRI mice compared to the HFD IRI mice, suggestive of a protective effect of NAC in the HFD group.

LLS was then evaluated for differences in lipid oxidation between the samples. There was a noticeable increase in LLS in the HFD, as seen by the increasing red color of images, as compared to the ND mice. We then quantified this fractional intensity of LLS (**Figure 3D**), as described in the supplemental methods. HFD and HFD IRI mice both demonstrate statistically significant higher quantifications of LLS compared to ND and ND IRI mice, respectively.

### LLS imaging demonstrates a reduction of lipid droplet size in HFD + NAC mice, which corresponds to a significant reduction of triacylglycerols

**Figure 4:**
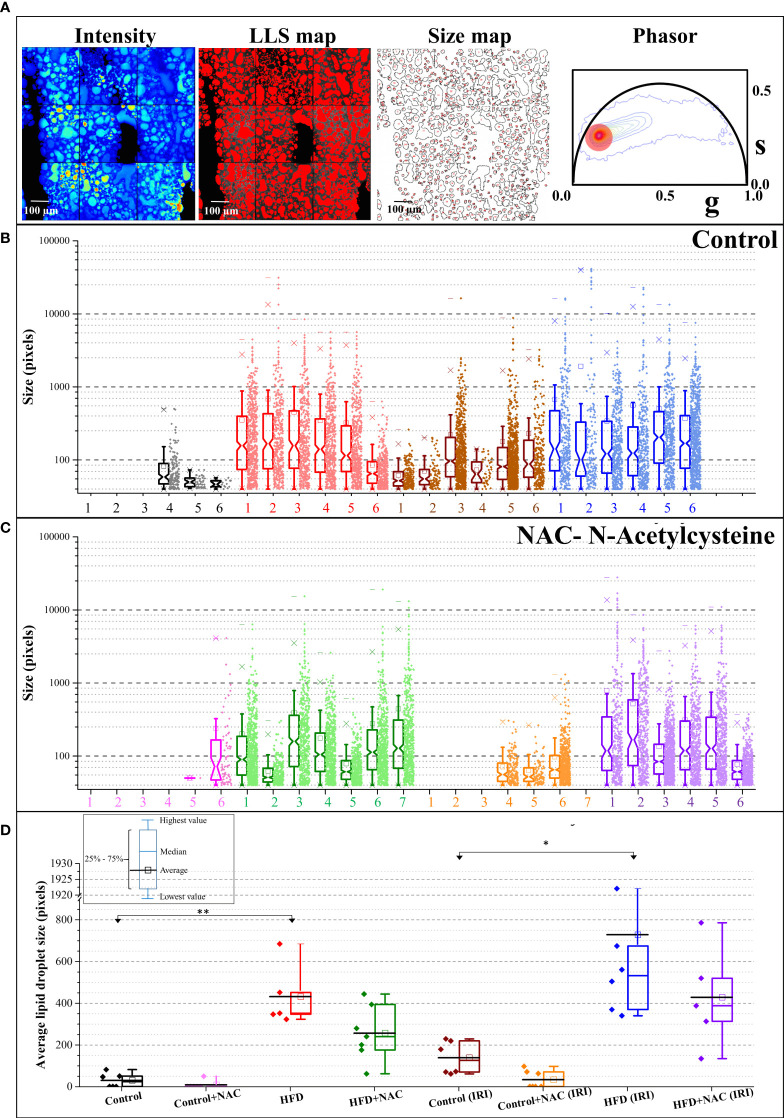
NAC treatment significantly reduced hepatic steatosis in HFD mice. **(A)** The fluorescence intensity image, the LLS mapped image, the calculated size distribution maps, and the corresponding phasor plot. The red cursor was used to select the LLS lifetime signature originating from the intensity image and the LLS image was colored according to that phasor signature. The size map was created to show the distribution of the droplets. **(B, C)** The size map was created to show the distribution of the droplets. **(B, C)** The size distribution plots for Control **(B)** and NAC **(C)** treatment. The Y axis is shown in logscale to exemplify the increasing size as a function of diet and IRI. The distribution shows HFD and IRI induce the large lipid droplets and (especially above 10000 pixels) are lower in number in HFD + NAC, groups. (n=5-7 mice per group). **(D)** The average size distribution of lipid droplets from the livers of the animals in different feeding regimen. “*” 0.05 to 0.01, “**”0.01 to 0.001.

Upon establishing that a higher degree of IRI is present on a background of hepatic steatosis, as demonstrated by biochemical, MRI, and glycolytic analysis, we next sought to quantify hepatic steatosis more accurately within our samples. Phasor FLIM imaging allows us to quantifiably calculate steatosis based on the accumulation of the LLS (27). Selection of the phasor signature of LLS (long lifetime species, red circle in **Figure 4A**), enables the identification of the lipid droplets in autofluorescence FLIM images, where they are mapped according to the color of the selection (red here). Steatosis is then quantified by calculating the size of the droplets occupying the image. Our data show that droplets are much smaller or are nonexistent in ND diet mice and are much larger in HFD diet mice, both in the IRI and sham samples, which correlates to pathological evaluation of H&E staining. Note that the Y axis is in log scale and shows that exceptionally large droplets are seen in HFD livers in both sham and IRI mice as compared to ND mice. Importantly, while the average size of the lipid droplets increases in both HFD and HFD IRI mice, this is visibly reduced in HFD + NAC and HFD + NAC IRI mice (**Figures 4B–D** **)**. It is further notable that, while not statistically significant, there is a visible and quantifiable increase in lipid droplet size in ND IRI and HFD IRI mice when compared to the respective ND and HFD counterparts. It is important to note that upon evaluation of the individual distributions of lipid droplet within each sample (**Supplemental Figure 4**), it demonstrated that a large degree of variation in lipid droplet sizes is seen in each sample.

**Figure 5:**
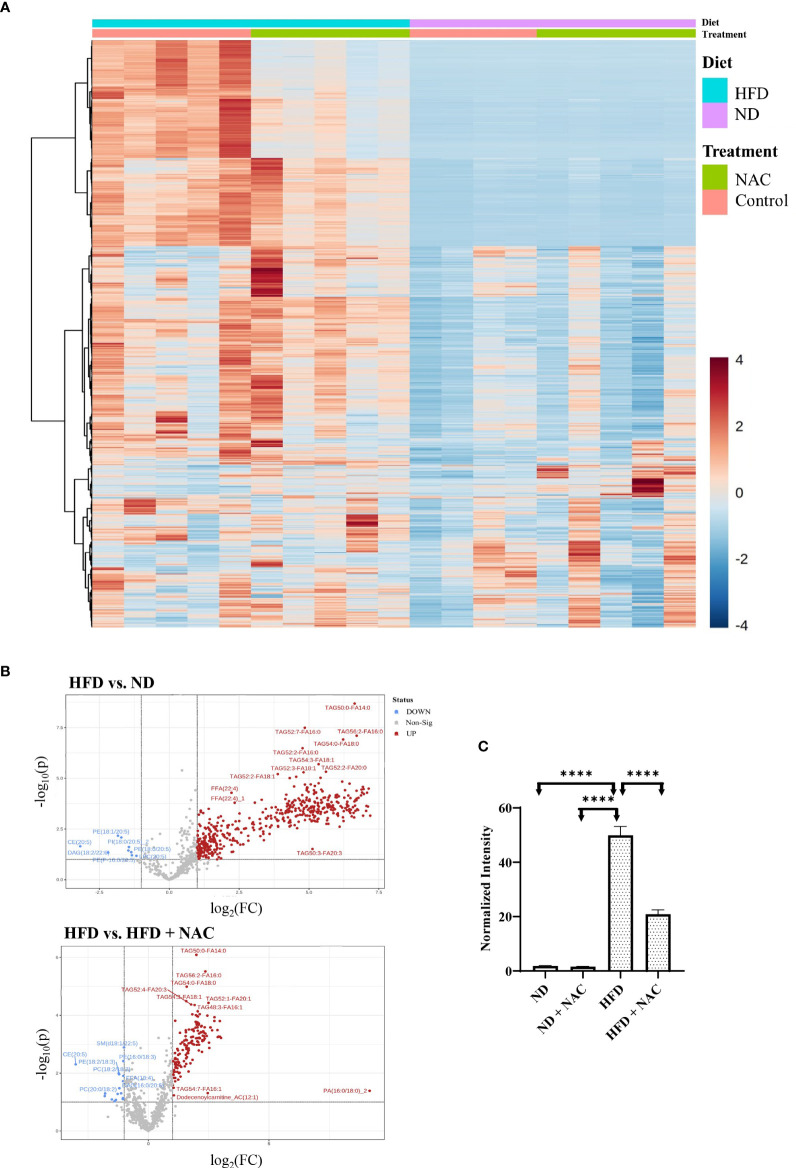
NAC treatment in HFD mice significantly altered the lipid profile within the liver. **(A)** Heat map demonstrating the alterations in lipid composition with the respective tissue. **(B)** Volcano plots demonstrating the differences in upregulated lipid species in HFD *vs.* ND and HFD *vs.* HFD + NAC mice, ultimately revealing changes TAG composition within these tissues. **(C)** Histogram representing quantitative differences in TAG composition between the treatment groups. “****” <0.0001.

Prepared snap-frozen liver tissue from sham mice was subsequently subjected to metabolomic profiling of 21 classes of lipid metabolites. We identified significant changes in lipid composition between the tissues, as visible in the heat map (**Figure 5A**). Of the 21 classes analyzed, triacylglycerols (TAG) were significantly upregulated in HFD mice as compared to normal diet (0.222 normalized intensity *vs*. 6.783 normalized intensity, p<0.0001). This was significantly decreased in the HFD + NAC mice, as demonstrated in the volcano plots showing differences in upregulated lipid profile in HFD *vs.* ND and HFD + NAC *vs.* HFD mice (**Figure 5B**). Interestingly, the HFD + NAC mice had a statistically reduced composition of TAG, as compared to HFD mice (6.281 normalized intensity *vs*. 2.681 normalized intensity, p<0.0001; **Figure 5C**). The reduction of TAG species demonstrated with NAC supplementation was largely global, affecting 149 of the 277 species evaluated. There were no statistically significant reductions in the HFD + NAC mice for the remaining 20 classes of lipids.

### IRI results in specific genetic upregulation in HFD mice

**Figure 6:**
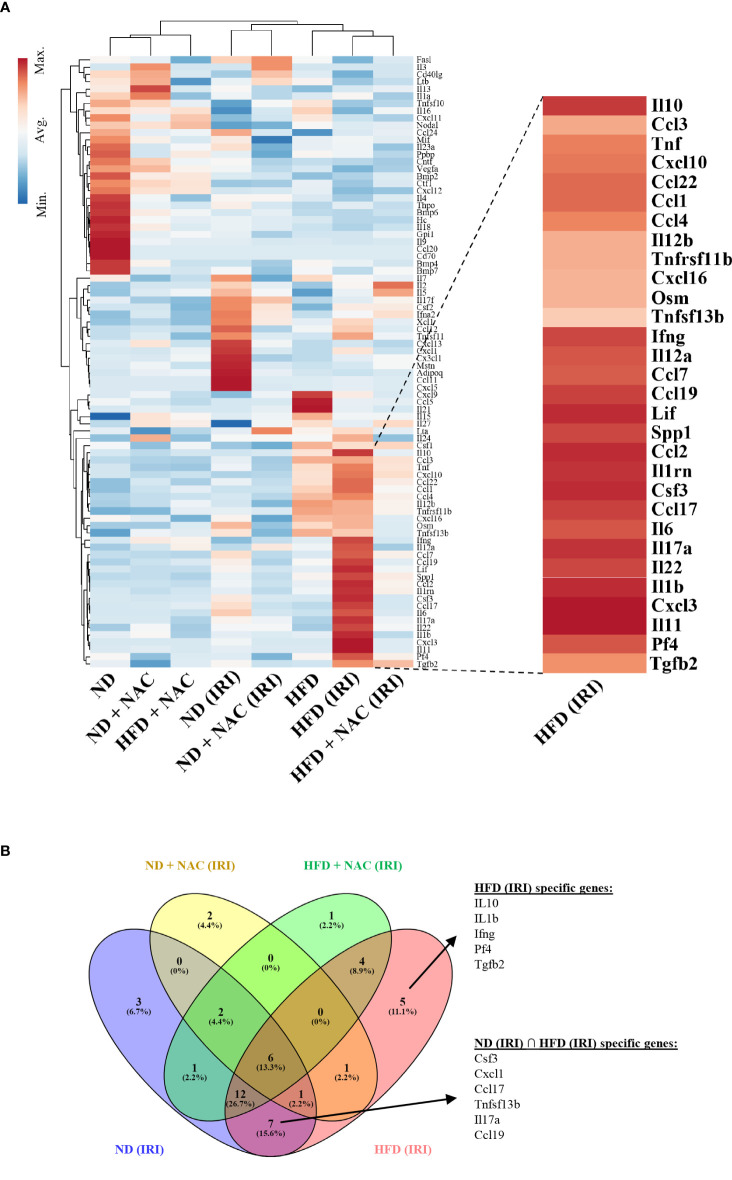
IRI induced specific genetic upregulation in HFD mice. RT^2^ profiler PCR array for cytokines and chemokines was utilized to examine differences in genetic regulation among treatment groups. Each group represents a single pooled sample from at least three separate liver-tissue specimens. **(A)** Heat map of cytokine and chemokine PCR analysis. **(B)** Venn Diagram demonstrating genes that are upregulated in HFD (IRI) and those shared amongst ND (IRI) and HFD (IRI).

In order to identify specific transcriptomic changes that occur in the liver following IRI, we performed array-based PCR transcriptional profiling using the RT^2^ profiler PCR array for 84 distinct Chemokine and Cytokines (Qiagen, Germantown, MD). Utilizing an unsupervised hierarchical clustering analysis, we demonstrated a distinct difference in gene expression patterns between HFD mice and those mice that underwent IRI (**Figure 6A**). We then identified both shared and discrete genes with a minimum of two-fold upregulation amongst the mouse groups that underwent IRI (**Figure 6B**).

We identified 45 genes of interest that were upregulated within the collective specimens. Among these genes, seven were uniformly upregulated in both ND IRI and HFD IRI groups, including *CSF3, CXCL1, CCL17, CCL19, TNFSF13b* (TNF Superfamily Member 13b), *IL-6* and *IL17a*. Uniquely, HFD IRI mice exhibited an exclusive upregulation of five genes: *TGFβ-2, Pf4* (Platelet Factor 4), *IL-1β, IL-10*, and *IFN-γ*. In comparison, HFD + NAC mice that underwent IRI exhibited significantly lower expression levels of the genes that were exclusively upregulated in HFD IRI and shared between ND IRI and HFD IRI mice.

### IFN-γ producing CD3+/NK1.1+ and CD3+/CD1d+ cells drastically contribute to hepatic IRI and are mitigated with NAC treatment

Given the significant upregulation of IFN-γ and its related genes shown by RT-PCR array analysis and the variations in lipidomic analysis, specifically TAGs, we next aimed to specifically evaluate the role of IFN-γ producing NKT cells in hepatic IRI by polychromatic flow cytometry. We first evaluated this using NKT1 cell surrogate markers defined as CD45+/CD3+/NK1.1+ cells. Utilizing this strategy, we first evaluated the sham mice from each group. ND mice harbored NKT cells at an average frequency of 13.65%. This was significantly higher than the HFD mice (6.97%, p=0.004). Interestingly, while there was no significant difference between the HFD and HFD + NAC groups, the ND + NAC mice were shown to harbor a larger frequency of CD45+/CD3+/NK1.1+ cells as compared to ND mice (27.5%, p=0.017).

**Figure 7:**
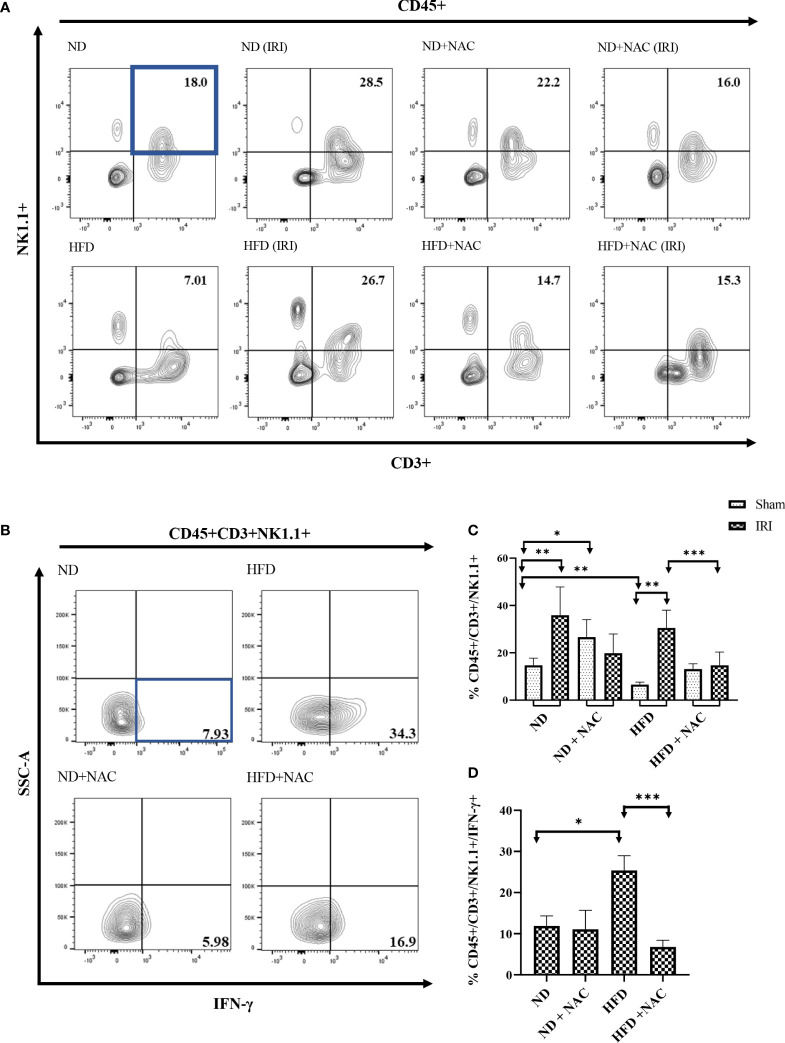
IFN-γ producing Hepatic CD3+/NK1.1+ cells are contributors following IRI and successfully ameliorated with NAC supplementation. **(A)** Representative contour plots of the percentage of CD45+/CD3+/NK1.1+ NKT1 cells within Hepatic Mononuclear cells. Number of mice per group is as follows: ND=6, ND (IRI)=5, ND + NAC= 5, ND + NAC (IRI)=11, HFD=5, HFD (IRI)=5, HFD + NAC=5, HFD + NAC (IRI)=10. **(B)** Representative contour plots of the percentage of IFN-γ producing CD45+/CD3+/NK1.1+ NKT cells after IRI. Number of mice per group is as follows: ND (IRI)=5, ND + NAC (IRI)=5, HFD (IRI)=7, HFD + NAC (IRI)=10. **(C)** Histogram demonstrating the percentage of NKT1 cells illustrated in **(A)**. **(D)** Histogram demonstrating the percentage of NKT1 cells illustrated in **(B)**. “*” 0.05 to 0.01, “**”0.01 to 0.001, “***” 0.001 to 0.0001.

We then elicited hepatic IRI and evaluated for CD45+/CD3+/NK1.1+ cell changes. ND IRI mice had a significant rise in the percentage of CD45+/CD3+/NK1.1+ cells as compared to the ND sham mice (p=0.0095). There were no significant differences in NKT cells frequencies amongst ND + NAC IRI mice or the ND + NAC sham mice, despite the increased frequencies in ND + NAC mice, as previously demonstrated. Interestingly, following reperfusion injury, HFD IRI mice underwent a drastic rise in CD45+/CD3+/NK1.1+ cells as compared to HFD sham mice (28.9%, p=0.0079), which was statistically similar to that seen in the ND IRI group (**Figures 7A, C** **)**. This effect was ameliorated following NAC treatment in HFD mice. The HFD + NAC IRI mice showed no significant differences in NKT cell populations as compared to the HFD SHAM counterparts; however, the frequency of NKT cells was drastically reduced when compared to both HFD IRI and ND IRI groups.

We next employed *ex vivo* cell stimulation with PMA from samples obtained from all IRI groups to identify the level of IFN-γ and TNF-α produced from CD45+CD3+ NK1.1+NKT cells, as demonstrated in **Supplemental Figure 5**. While the level of IFN-γ produced was similar between ND IRI and ND + NAC IRI mice, there was a significantly greater frequency of IFN-γ produced by HFD IRI mice (**Figures 7B, D** **)**, corresponding to the previously demonstrated cell populations (p=0.0177). This rise in IFN-γ production was ameliorated in the HFD + NAC IRI mice as compared to the HFD IRI mice (p=0.0001). The levels of TNF-α were significantly higher in the ND IRI than the HFD IRI mice, and there was no significant difference between HFD IRI and HFD + NAC IRI mice (**Supplemental Figure 6**).

**Figure 8:**
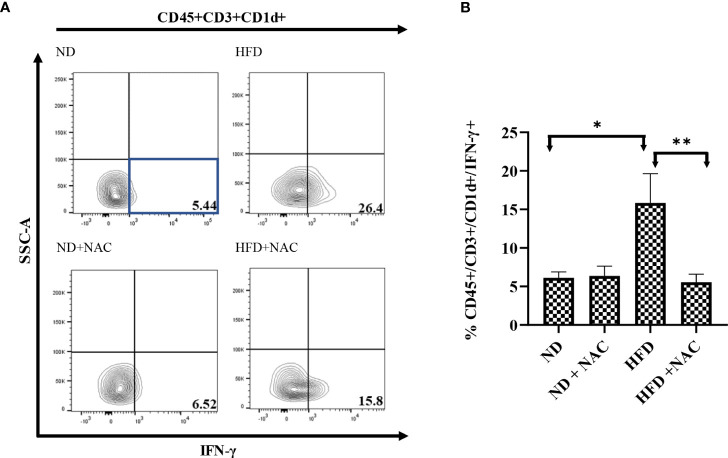
IFN-γ producing hepatic CD3+/CD1d+ cells are contributors following IRI and successfully ameliorated with NAC supplementation. **(A)** Representative contour plots of the percentage of IFN-γ producing CD45+/CD3+/CD1d+ NKT cells after IRI. Number of mice per group is as follows: ND (IRI)=5, ND + NAC (IRI)=5, HFD (IRI)=7, HFD + NAC (IRI)=10. **(B)** Histogram demonstrating the percentage of IFN-γ producing CD45+/CD3+/CD1d+ NKT cells illustrated in **(A)**. “*” 0.05 to 0.01, “**”0.01 to 0.001.

Finally, we validated our findings by evaluating the effect of NAC supplementation on the IFN-γ production from CD1d bound NKT cells (CD45+/CD3+/CD1d+ cells). HFD IRI mice had a significantly higher frequency of IFN-γ from CD45+/CD3+/CD1d+ cells as compared to ND IRI mice (p=0.017). This effect was significantly reduced in the HFD + NAC IRI mice (p=0.0012) (**Figures 8A, B** **)**. There were no significant differences in TNF-α production from these cells in the HFD IRI and HFD + NAC IRI mice (**Supplemental Figure 6**).

## Discussion

Nonalcoholic Fatty Liver Disease (NAFLD) remains a significant problem in liver transplantation. Not only can NAFLD progress to cirrhosis/liver failure (44) and contribute to the development of hepatocellular carcinoma (both indications for liver transplantation), but it also affects the potential donor population. In this regard, steatotic allografts experience a greater degree of IRI upon reperfusion, which increases the risk of poor clinical outcomes in decompensated recipients. A key component towards increasing the potential donor population involves effectively preventing, diagnosing, and treating IRI. As such, reliable animal models for the study of hepatic steatosis and the innate immune system response to IRI are paramount. In this work, we demonstrated the effectiveness of a HFD murine model and primary treatment group to highlight novel and noninvasive methods of investigating liver injury, as well as identify a key immune regulatory strategy in the prevention of IRI.

The efficacy of a diet induced-obesity murine model is well established (1, 45). However, potential therapeutics are limited. Intravenous (IV) NAC has previously been successful in the treatment of IRI, both clinically and experimentally, with a well-established role in the reduction of reactive oxygen species (2, 46–48). Recently, oral NAC supplementation was investigated for its use in the treatment of metabolic disorders and was shown to decrease hepatic steatosis *via* a PPAR-γ dependent mechanism and stimulation of thermogenic gene expression (45). Our work has expanded upon these findings, as we have demonstrated that oral NAC treatment decreased hepatic steatosis and increased BAT as detected by T1 weighted MRI. In this sense, BAT is characteristic of high metabolism and thermogenic capacity (49), which supports that NAC alters thermogenic gene expression. We were further able to correlate the decreased hepatic steatosis with improved liver functional capacity using Eovist^®^ DCE MRI, which has emerged as a useful hepatobiliary contrast agent that clinically, and non-invasively, allows for stratification of steatotic liver damage (50). We demonstrated a stark difference in contrast uptake in HFD mice in comparison to the ND mice, which was normalized with NAC supplementation, especially at 18 and 30 minutes post-injection of Eovist^®^. While the mechanisms by which NAC improves Eovist^®^ uptake in the HFD mice are presently unknown, previous work in cirrhotic rats suggests alterations in the expression of associated membrane proteins may be a key factor (51), although alternations in hepatic perfusion cannot be ruled out and additional studies are warranted to establish these relationships. Overall, these findings collectively established this HFD model with NAC treatment group as an effective method to study IRI mitigation.

Despite the known effect of IV NAC in IRI, to our knowledge, this is the first study to address the effectiveness of oral NAC supplementation in the prevention of IRI. Utilizing our clinically relevant model, we demonstrated improvement of IRI in HFD mice on NAC supplementation as compared to HFD only using serum ALT and T2 MRI mapping. Interestingly, T2 relaxation times, which correlate with hepatic edema and inflammation (52), show a similar effect in all lobes of the liver. This demonstrates the scope of full liver sequelae following segmental injury in HFD mice, which is significantly reduced in HFD + NAC mice. Previous work has demonstrated the vasodilatory effects of NAC in models *via* Nitric Oxide dependent (46, 47) and Vascular Endothelial Growth Factor (VEGF)/VEGFR2 (47, 48) dependent mechanism, which may offer insight into the reduced edema visualized in our samples. However, this does not provide insight into the key roles of innate immune system cytokine generation and metabolic changes at a cellular level which occur following IRI.

While it is known that NAFLD is associated with significant modulation of metabolic pathways involved in glycolysis (53, 54) and lipid oxidation (55), the alterations in these pathways following IRI are the subject of ongoing research. To elicit some insight into this area, we employed Phasor-FLIM and SHG imaging as a novel approach in the evaluation of hepatic IRI, where we demonstrated increased glycolytic stress and lipid oxidation within our HFD murine model that was worsened following IRI. This is consistent with known requirements of NADPH and FAD metabolism (43). We further determined that NAC supplementation decreased glycolytic stress in HFD mice following IRI, correlating to a less severe degree of injury and inflammation that was corroborated by our previous findings in serum ALT and T2 mapping. Interestingly, we did not appreciate the same changes in lipid oxidation. Additionally, utilizing LLS imaging of lipid droplets, we further appreciate a visual reduction in lipid droplet size in our HFD + NAC mice when compared to HFD mice. However, when an average value was calculated for total image, this resulted in loss of dimensional information and the inherent, visually depicted heterogeneity (**Supplemental Figure 4**). The actual distribution of the fractional intensity of LLS (red) and free NADH (blue) demonstrate that embedded spatial information is a key component phasor-FLIM auto fluorescent image analysis. This is demonstrated in the composite image of LLS, free and bound NADH and the individual species distribution (**Supplemental Figures 4C-F**), which demonstrates how different parts of the image have a differential amount of each species. The cumulative graphs from 3 samples show that along with a shift of LLS in HFD IRI samples, there may also be broadening of these distributions. In case of broadening, e.g. HFD + NAC *vs*. ND – the central position of the distribution may not shift appreciably, however more metabolic heterogeneity is seen in the HFD + NAC samples than in the ND diet samples, which is apparent. Further, while previous work had shown a downregulation of genes involved in lipid oxidation in obesity induced mice on NAC supplementation (45), this was not apparent on LLS imaging for lipid oxidation. Importantly, LLS is a complex and incompletely understood representation of lipid oxidation byproducts that depends greatly on the different pathways of oxidation. Thus, absence of changes in LLS does not confidently correlate to a lack of lipid oxidation throughout the tissue (56), which is the subject of ongoing research.

To further classify metabolic changes occurring with NAC supplementation, we performed a quantitative lipid profile analysis of whole liver tissue. To our knowledge, this is first time a full lipidomic profile has been generated from liver tissue of HFD mice on NAC supplementation. This demonstrated significant downregulation of TAGs species when compared to mice on HFD alone. TAG synthesis is known to be regulated *via* a PPAR-γ dependent pathway (57), and previous studies have demonstrated that PPAR-γ activity is downregulated as a result of NAC supplementation (45, 58). Recently, PPAR-γ has also been identified as a key mediator in the synthesis of CD1d (59), which serves a crucial function in priming of NKT cells. This expands on work that previously demonstrated NKT cell activation occurs secondary to lipid excess (7, 14, 60), which has been linked to CD1d expression on adipocytes. It has further been demonstrated that NKT cell activation is secondarily decreased in *ApoE ^-/-^* mice (61). Further work by Downs et al. demonstrated that NAC supplementation can abolish Vα14iNKT cells and IFN-γ signaling in fulminant liver failure (62). Taken together, our findings further suggest that NAC treatment could prevent NKT cell activation by preventing lipid excess and downregulating CD1d.

Our subsequent experiments were formulated around determination of NKT cell activity. RT-PCR array for cytokine and chemokine expression was performed from liver tissue in the maximally affected left lobe. This ultimately eluded to significant upregulation in HFD IRI mice that was not established elsewhere, specifically IFN-γ and its related genes, including IL-12. Upon further analysis, we appreciated significantly upregulated in genes that have been linked to the NF-κB pathway—CXCL1 (63, 64) and CXCL10 (63, 65). This is in line with previous reports that the NAC treatment results in a downregulation of NF-κB (45, 66), and also furthered our suspicion that NKT cells were in fact mediators in the model, as IFN-γ (67) and, more specifically, TCR-β both result in direct activation of the NF-κB pathway (68, 69). Further, IFN-γ also results in NF-κB activation by means of JAK/STAT activation pathways. Finally, we elicited a complete downregulation of these associated genes in HFD + NAC IRI mice. This ultimately alludes to an injurious effect of IFN-γ that can be, at least partially, alleviated by NAC supplementation. However, we cannot fully exclude other mechanisms, and additional studies will be needed to confirm our findings.

While our flow cytometry findings suggest that NKT cell activation and cytokine production are affected by NAC, additional studies using a CD1d deficient mouse model and adoptive transfer experiments of IFN-gamma-deficient NKT cells into *Ja18^-/-^* mice are needed within our HFD NAC model for definitive confirmation. Moreover, we observed a significant upregulation of IL-17 within our HFD IRI mice that was subsequently ameliorated by NAC. We have previously demonstrated that IL-17 produced by unconventional RORγt T-cells contribute to hepatic ischemia reperfusion injury (70). More recently, IL-17 producing NKT cells have been described and implicated in inflammatory diseases (71) and may also play a critical role in IRI. Further comparative studies will be needed to identify the role of NAC in IL-17 reduction and the degree of injury prevention, as compared to IFN-γ.

Overall, our work utilized novel methods of hepatic injury identification to demonstrate the effectiveness of oral NAC supplementation in preventing IRI. We further identified potential downstream pathways and targets for future research into the prevention of hepatic IRI. Further work will be required to definitively establish mechanism behind the protective effect of oral NAC supplementation.

## Conclusion

Oral N-acetylcysteine supplementation can ameliorate IRI in a HFD mouse model. IFN-γ pathways and NKT cells are largely implicated in this observed effect.

## Data availability statement

The original contributions presented in the study are publicly available. This data can be found in NCBI's Gene Expression Omnibus; GEO accession number: GSE209733. Further inquiries can be directed to the corresponding authors.

## Ethics statement

The animal study was reviewed and approved by Georgetown University Institutional Animal Care and Use Committee.

## Author contributions

JL, JK, and SR equally contributed experiment completion, data analysis, authorship and critical review of this manuscript. AK, JL, JK, SR, OR, KL, DP, YC, KO, BF, SN, BH, YL, DK, WC, KK, HL, and BK acquired, analyzed and/or critically reviewed data. AK, SCR, KK, WC, CA, TF, SR, and ML secured funding, contributed to study design and facilitated critical oversight of the project. All authors contributed to the article and approved the submitted version.

## Funding

Funding was provided by NIH R21AI130800 (AK, SCR), with contributions from NIH S10 OD025153 (CA), NIH-P30 CA051008-22 (CA), NIH/NCI P30-CA051008 (Microscopy & Imaging Shared Resource), NIH R01 DK116567 and R01DK127830 (SR and ML), and private funding from the Children’s Rare Disease Organization Inc. (AK, JK, KK).

## Acknowledgments

Gadoxetate Disodium (Eovist^®^/Primovist^®^) was kindly provided by BayerHealthCare *AG, D-51368 Leverkusen, Germany* (CA). The CD1d Tetramer was graciously provided by the National Institute of Health Core Tetramer Facility, Atlanta, GA.All *in vivo* imaging was carried out in the Georgetown-Lombardi Preclinical Imaging Research Laboratory and all flow cytometry analyses were performed in Flow Cytometry & Cell Sorting Shared Resource.

## Conflict of interest

Georgetown University filed a patent related to this manuscript. KK, WC, and AK are named as inventors on this application and declare that as a potential conflict of interest. The remaining authors declare that the research was conducted in the absence of any commercial or financial relationships that could be construed as a potential conflict of interest.

## Publisher’s note

All claims expressed in this article are solely those of the authors and do not necessarily represent those of their affiliated organizations, or those of the publisher, the editors and the reviewers. Any product that may be evaluated in this article, or claim that may be made by its manufacturer, is not guaranteed or endorsed by the publisher.

## Author’s disclaimer

The views expressed in this manuscript reflect the results of research conducted by the authors and do not necessarily reflect the official policy or position of the Department of the Navy, Department of Defense, or the United States Government.

JL is a military service member. This work was prepared as part of official duties. Title 17, USC, Section 105 provides that Copyright protection under this title is not available for any work of the U.S. Government and defines a U.S. Government work as a work prepared by a military service member or employee of the U.S. Government as part of that person’s official duties.
