# Supplementary material for: Oral N-acetylcysteine decreases IFN-γ production and ameliorates ischemia-reperfusion injury in steatotic livers
Source: Front Immunol. 2022 Sep 5;13:898799. doi: 10.3389/fimmu.2022.898799 (PMC9486542; doi:10.3389/fimmu.2022.898799)
Supplement: Supplementary file 1 [file DataSheet_1.pdf]

Oral N-acetylcysteine decreases IFN-γ production and ameliorates ischemia-reperfusion injury in steatotic livers.

Jedson R. Liggett[1,2†], Jiman Kang[1,3†], Suman Ranjit[3,4*†], Olga Rodriguez[5,6], Katrina Loh[1], Digvijay Patil[1], Yuki Cui[1], Anju Duttargi[6], Sang Nguyen[5], Britney He[5], Yichien Lee[5,6], Kesha Oza[1], Brett S. Frank[1], DongHyang Kwon[7], Heng-Hong Li[6], Bhaskar Kallakury[7], Andrew Libby[8], Moshe Levi[3], Simon C. Robson[9], Thomas M Fishbein[1], Wanxing Cui[1,3], Chris Albanese[5,6,10], Khalid Khan[1], Alexander Kroemer[1*]

[1] MedStar Georgetown Transplant Institute, MedStar Georgetown University Hospital and the Center for Translational Transplant Medicine, Georgetown University Medical Center, Washington, District of Columbia, USA.
[2] Department of Surgery, Naval Medical Center Portsmouth, Portsmouth, Virginia, USA.
[3] Department of Biochemistry and Molecular & Cellular Biology, Georgetown University, Washington, District of Columbia, USA.
[4] Microscopy & Imaging Shared Resource, Georgetown University, Washington, District of Columbia, USA.
[5] Center for Translational Imaging, Georgetown University Medical Center, Washington, District of Columbia, USA.
[6] Department of Oncology, Lombardi Comprehensive Cancer Center, Georgetown University Medical Center, Washington, District of Columbia, USA.
[7] Department of Pathology, MedStar Georgetown University Hospital, Washington, District of Columbia, USA.
[8] Division of Endocrinology, Metabolism, & Diabetes, University of Colorado Anschutz Medical Campus, Aurora, Colorado, USA.
[9] Departments of Anesthesiology and Medicine, Beth Israel Deaconess Medical Center, Harvard Medical School, Boston, Massachusetts, USA.
[10] Department of Radiology, MedStar Georgetown University Hospital, Washington, District of Columbia, USA.

Supplementary Materials

1.0 SUPPLEMENTAL METHODS: Fluorescence lifetime imaging

1.1 Instrumentation:

The autofluorescence lifetime images of the liver samples were measured using a modified Olympus FVMPERS (Waltham, MA) microscope equipped with a Spectra-Physics Insight X3 (Milpitas, CA) laser and FastFLIM (ISS, Champaign, IL) acquisition card. The samples were excited with the 740 nm laser line using a 20X air objective (LUCPLFLN 0.45NA, Olympus) applying a two-photon excitation scheme. The fluorescence was collected using the DIVER (Deep Imaging Via Enhanced Recovery) detector assembly and recorded using a FastFLIM card. The pixel dwell time for the acquisitions was 20 µs and the images were taken with sizes of 256 x 256 pixels with a field of view of 318.8 µm (Zoom =2X). To have a high signal-to-noise ratio, 16 frames were collected for each area. The data from each pixel were recorded and analyzed using the SimFCS software (Laboratory for Fluorescence Dynamics, University of California, Irvine, CA). The FLIM data were collected using the passive mode, where the raster scanning was done using the Olympus software and the images were collected using the FLIMBox/FastFLIM system, and the scanning parameters were matched to ensure proper image acquisition.

1.2 DIVER detection:

The autofluorescence of liver sections (5 µm thick) was imaged using the homebuilt DIVER (Deep Imaging via Enhanced Recovery) microscope (1-3). DIVER is a homebuilt modified detector based on an upright configuration. The details of this microscope have been described elsewhere (1-4). Briefly, this microscope uses a forward detection scheme that is ideally suited for imaging at shorter wavelengths and has higher photon collection efficiency due to the large cone angle of detection. The large area photon counting detector (R7600P-300, Hamamatsu) of the DIVER is connected to a FastFLIM unit (ISS, Champaign, IL) which can acquire the fluorescence decays at each pixel of the image. The sample was placed on top of the detector assembly consisting of the housing with filter wheel and large area PMT. An index matching liquid in the microscope optical path from the sample to the detector prevents the loss of photons by reflections. The signal from the tissue sample is passed through the filter wheel and is collected by the PMT in the direction of excitation. Two BG39 filters create the incoming and outgoing window of the filter assembly and prevent the excitation laser from hitting the detector. A combination of a filter capable of separating the blue wavelength (400 – 500 nm) fluorescence was used for FLIM imaging of NADH(4). The phasor plot is calibrated using Rhodamine 110 in water which has a mono-exponential lifetime of 4.0 ns.

1.3 <u>Phasor Analysis (7, 8):</u>

The fluorescence intensity decays collected at each pixel of the image were transformed to the Fourier space and the phasor coordinates were calculated using the following relations:

$$g_{i,j}(\omega) = \int_0^\infty I(t)\cdot\cos(n\omega t)dt / \int_0^\infty I(t)dt \qquad 1$$

$$s_{i,j}(\omega) = \int_0^\infty I(t)\cdot\sin(n\omega t)\,dt / \int_0^\infty I(t)dt \qquad 2$$

where, $g_{i,j}(\omega)$ and $s_{i,j}(\omega)$ are the X and Y coordinates of the phasor plot, respectively, and n and ω are the harmonic number and the angular frequency of excitation, respectively. The transformed data was then plotted in the phasor plot in a way that the data from each individual pixel is transformed to a point in the phasor plot (6-9).

1.4 Phasor Analysis for multiple components (6-11):

In frequency-domain measurements, the transformation to the phasor plot uses the following relations,

$$g_{i,j}(\omega) = m_{i,j}\cdot\cos(\phi_{i,j}) \quad 3$$

$$s_{i,j}(\omega) = m_{i,j}\cdot\sin(\phi_{i,j}) \quad 4$$

where, $g_{i,j}(\omega)$ and $s_{i,j}(\omega)$ are again the X and Y coordinates of the phasor plot, respectively, and $m_{i,,j}$ and $\phi_{i,j}$ are the modulation and phase at the image pixel *i, j*. The longer lifetime is represented by the larger phase angle in the phasor plot (Supplemental Figure SM1). The distribution of phasor points originating from FLIM measurements appears on (for mono-exponential decays) or inside

(for multi-exponential decays) the universal circle (Supplemental Figure. SM1). The linear combinations between two components appear along the line joining the phasor positions on the universal semi-circle (red, dark green and blue, right panel Supplemental Figure SM1) are shown by the black lines. If each component has a multi-exponential decay, its location will be represented by phasors not in the universal circle, but the principle of linear combination remains valid. This is shown by the phasor positions whose decays involve three components (Supplemental Figure SM1).

Mathematically, for a two-component system, this is described by the following:

For decays described by sum of exponentials (2 in the derivation below), the intensity decay can be expressed as,

$$I(t) = A_1 \cdot e^{-t/\tau_1} + A_2 \cdot e^{-t/\tau_2} \quad 5$$

The calculated phasor coordinates from this decay are given by,

$$g(\omega) = \left(A_1\tau_1 \frac{1}{1+(\omega\tau_1)^2} + A_2\tau_2 \frac{1}{1+(\omega\tau_2)^2}\right)/(A_1\tau_1 + A_2\tau_2) \quad 6$$

$$s(\omega) = \left(A_1\tau_1 \frac{\omega\tau_1}{1+(\omega\tau_1)^2} + A_2\tau_2 \frac{\omega\tau_2}{1+(\omega\tau_2)^2}\right)/(A_1\tau_1 + A_2\tau_2) \quad 7$$

These expressions can be simplified using the definition of fractional intensity:

$$f_n = A_n \cdot \tau_n / \sum_n A_n \cdot \tau_n \quad 8$$

The phasor coordinates of the mixture are given by,

$$g(\omega) = f_1 \frac{1}{1+(\omega\tau_1)^2} + f_2 \frac{1}{1+(\omega\tau_2)^2} \qquad 9$$

$$s(\omega) = f_1 \frac{\omega\tau_1}{1+(\omega\tau_1)^2} + f_2 \frac{\omega\tau_2}{1+(\omega\tau_2)^2} \qquad 10$$

For multiple exponential decays the law of phasor addition holds and thus,

$$g(\omega) = \sum_n f_n \cdot g_n(\omega) \qquad 11$$

$$s(\omega) = \sum_n f_n \cdot s_n(\omega) \qquad 12$$

Equations 11 and 12 together with the fraction normalization equation:

$$\sum_n f_n = 1 \quad 13$$

Equation 11-13 are the system of equations that are used in this paper to solve for the unknown fractions f_n (10-13).

Equation 11-13 are the representation of the linear addition properties of phasor plot. According to this principle, if a pixel of an image has multiple components, then the position of the corresponding phasor point is inside the polygon whose vertices are occupied by the phasor points originating from the individual components. The distance of the image phasor point from any of the vertices is reciprocal to the fractional intensity component of that particular component. The higher the fractional intensity contribution of that particular species towards the total intensity of the image pixel, the closer the phasor point of that image pixel is to the corresponding component phasor position. For a three-component system, an algebraic solution of this system exists and this allows the breakdown of a phasor cloud from an image to the individual fractional intensity components.

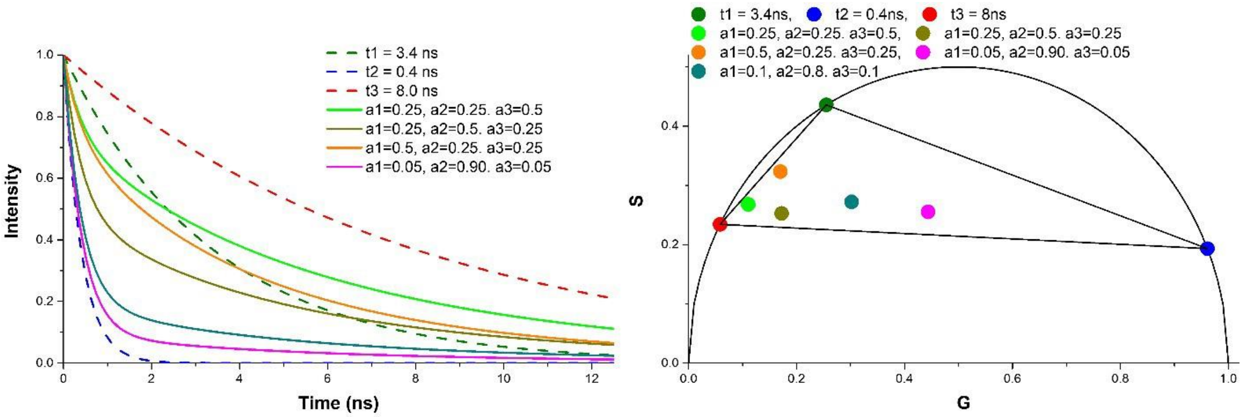

Supplemental Figure SM1. Calculated fluorescence intensity decays (left) and their phasor transformation (right). Mono-exponential decays are shown by dashed lines with red, dark green, and blue representing 8.0, 3.4, and 0.4 ns fluorescence lifetimes. The solid lines represent the decays calculated with different amounts of pre-exponential decays. The phasor plots of the corresponding decays are shown on the right with the same color scheme. The mono-exponential decays appear on the universal semi-circle (black) after the transformation and the

multicomponent decays appear within the triangle created by the phasor positions of the three mono-exponential decays. The red, dark green, and blue phasor positions are representative of the phasor positions of long lifetime species (LLS), LDH bound NADH (Lactose dehydrogenase), and free NADH, respectively.

1.5 Selection of the phasor positions of the autofluorescence species:

The three original phasor positions were selected based on one previously published work. Free and protein-bound NADH have a lifetime of 0.4 ns and ~3.4 ns respectively (13,14), and their individual fluorescence intensity decay is described by a mono-exponential (14, 15). In Supplemental Figure SM1 and Figure 3B, the phasor positions of free and bound NADH are represented by the blue and the green cursors, respectively. They appear on the universal semicircle (Figure) as their intensity decays can be explained by mono-exponentials (13-16). The line joining the two cursors is named metabolic trajectory. The long lifetime species (LLS) has a lifetime of ~8ns and the corresponding phasor position is selected by the red cursor in Figure 3B (17). In presence of LLS, the autofluorescence FLIM signal in NADH channel results in phasor cloud inside the triangle whose vertices are occupied by the central positions of red, green, and blue cursors.

1.6 Fractional intensity calculation and modification to concentration ratios:

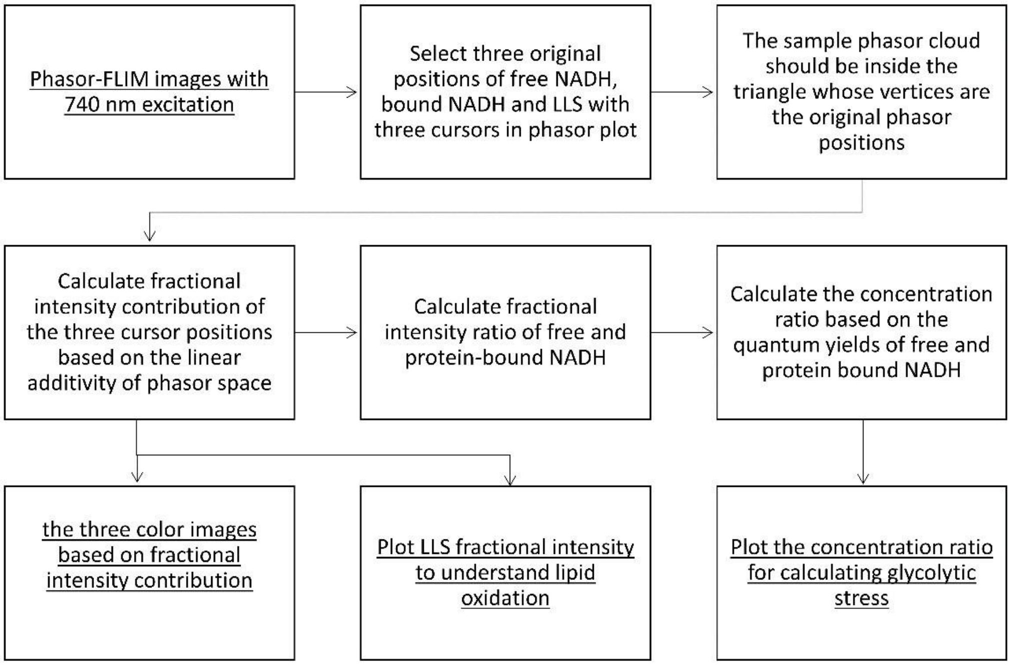

Supplemental Figure SM2. Calculation of the fractional intensity of LLS and calculation of glycolytic stress.

The phasor-FLIM images were obtained from the two-photon excitation induced by 740 nm and the emission was restricted to 400 – 500 nm. The resulting calibrated phasor plots were analyzed the following way for the fractional intensity contributions. First, the original phasor positions for free NADH, protein bound NADH and LLS were selected using published phasor positions of both of the species. The phasor plot from the liver samples resides within the triangle created by the phasor positions of these three components. The fractional intensity contributions of free NADH, bound NADH and LLS were calculated using the algebraic solution as published before (10-13). These fractional intensities were used along with relative color intensities to create the FLIM images. In these images, areas with more red, green, and blue shades are representative of more LLS, bound and free NADH, respectively. Exactly same color scheme was used to calculate all the images. The LLS fractional intensity from the liver from each of the animals were grouped for the type of the animal and diet and were plotted in a box plot showing the individual data points and the average value. To understand the glycolytic changes, the ratio of the fractional intensities of free and bound NADH were calculated, and then converted to the concentration ratio assuming the quantum yield of the two species (calculated from published results before). This conversion does not change the trend between the samples as this is a constant number (Supplemental Figure SM3). To do so – the quantum yield of free and bound NADH were calculated to be 0.03 and 0.45, respectively.

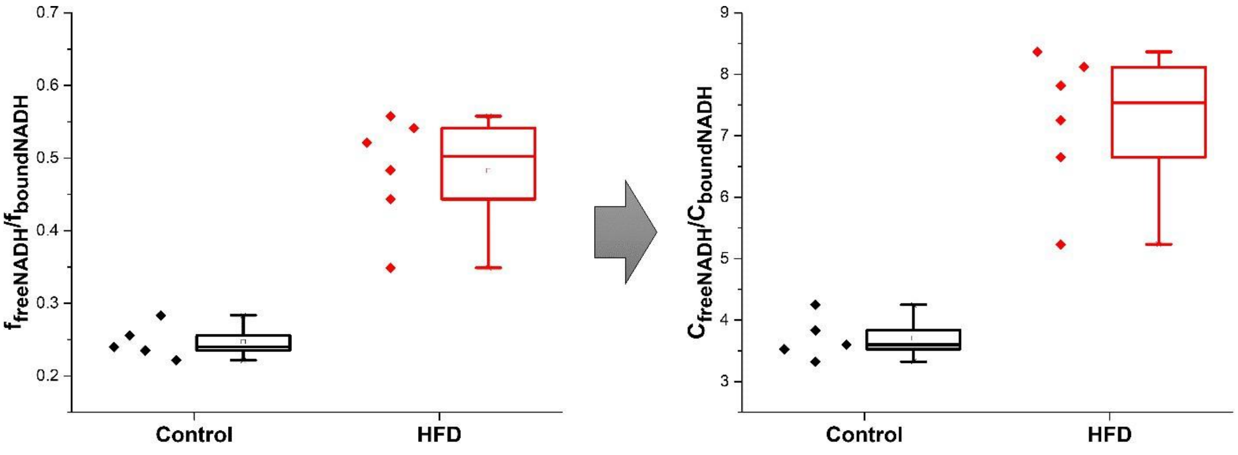

Supplemental Figure SM3. Conversion of fractional intensity ratios to concentration ratio. The fractional intensity ratio (left) was converted to the concentration ratio (right) by multiplication with the ratio of the quantum yields. The conversion does not change the relative changes measured by phasor-FLIM and only changes the scale.

1.7 Steatosis calculations:

Steatosis was calculated by the calculating the lipid droplets and then plotting the sizes (5, 15). The position of long lifetime species (LLS) in the phasor plot was selected with the red cursor and the FLIM images were colored accordingly. The LLS species is only present in the fat droplets and the size of the droplets can be measured by measuring the sizes of the areas of the images covered by LLS. To eliminate very small LLS areas – which can be caused by lower signal to noise ratio increasing the spread of phasor plot – a lower threshold value of 40 pixels were used to count areas covered by LLS. The details of the steps of the analysis is shown in Supplemental Figure SM4. The droplet sizes can then be represented by a box plot or by

histograms. The box plot shows how the average and standard deviation of the size of the droplets change based on the type of the diet. The average sizes from each animal were grouped together based on the diet and were plotted together to compare the changes between groups.

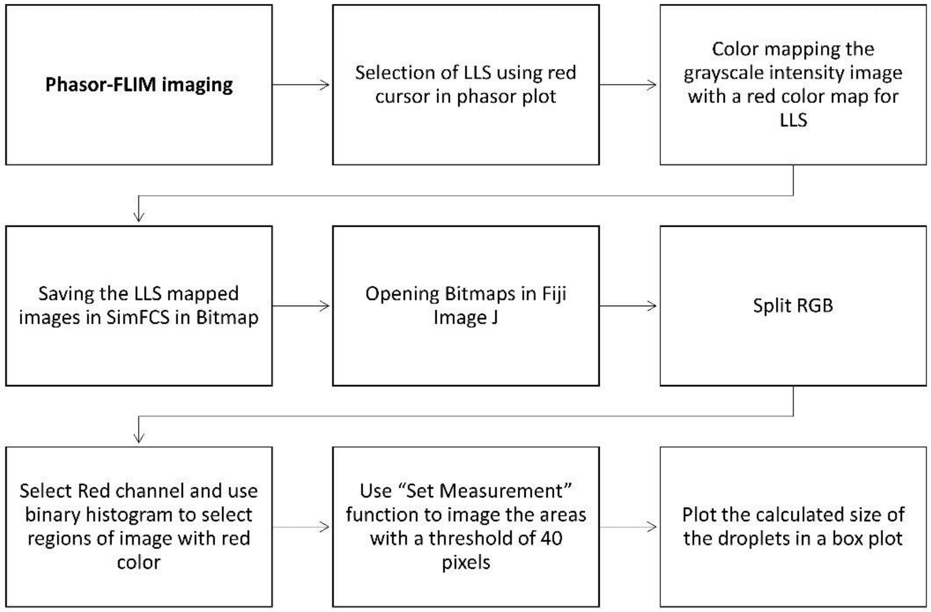

Supplemental Figure SM4. Calculation of steatosis based on phasor-FLIM of liver sample autofluorescence.

References:

1. Crosignani V, Dvornikov A, Aguilar JS, Stringari C, Edwards R, Mantulin WW, et al. Deep Tissue Fluorescence Imaging and in Vivo Biological Applications. *Journal of Biomedical Optics* 17(11):116023-.
2. Crosignani V, Jahid S, Dvornikov AS, Gratton E. A Deep Tissue Fluorescence Imaging System with Enhanced Shg Detection Capabilities. *Microscopy Research and Technique* 77(5):368-73.
3. Crosignani V, Jahid S, Dvornikov AS, Gratton E. A Deep Tissue Fluorescence Imaging System with Enhanced Shg Detection Capabilities. *Microsc Res Tech* (2014) 77(5):368-73. Epub 2014/03/07. doi: 10.1002/jemt.22354.
4. Ranjit S, Dvornikov A, Dobrinskikh E, Wang X, Luo Y, Levi M, et al. Measuring the Effect of a Western Diet on Liver Tissue Architecture by Flim Autofluorescence and Harmonic Generation Microscopy. *Biomed Opt Express* (2017) 8(7):3143-54. doi: 10.1364/BOE.8.003143.
5. Malacrida L, Ranjit S, Jameson DM, Gratton E. The Phasor Plot: A Universal Circle to Advance Fluorescence Lifetime Analysis and Interpretation. *Annu Rev Biophys* (2021) 50:575-93. Epub 2021/05/07. doi: 10.1146/annurev-biophys-062920-063631.
6. Digman MA, Caiolfa VR, Zamai M, Gratton E. The Phasor Approach to Fluorescence Lifetime Imaging Analysis. *Biophys J* (2008) 94(2):L14-L6. Epub 2007/11/02. doi: 10.1529/biophysj.107.120154.

7. Clayton AH, Hanley QS, Verveer PJ. Graphical Representation and Multicomponent Analysis of Single-Frequency Fluorescence Lifetime Imaging Microscopy Data. *J Microsc* (2004) 213(Pt 1):1-5. Epub 2003/12/18.
8. Redford GI, Clegg RM. Polar Plot Representation for Frequency-Domain Analysis of Fluorescence Lifetimes. *J Fluoresc* (2005) 15(5):805-15. Epub 2005/12/13. doi: 10.1007/s10895-005-2990-8.
9. Stringari C, Edwards RA, Pate KT, Waterman ML, Donovan PJ, Gratton E. Metabolic Trajectory of Cellular Differentiation in Small Intestine by Phasor Fluorescence Lifetime Microscopy of Nadh. *Sci Rep* (2012) 2:568. Epub 2012/08/15. doi: 10.1038/srep00568.
10. Torrado B, Malacrida L, Ranjit S. Linear Combination Properties of the Phasor Space in Fluorescence Imaging. *Sensors* (2022) 22(3):999.
11. Vallmitjana A, Torrado B, Dvornikov A, Ranjit S, Gratton E. Blind Resolution of Lifetime Components in Individual Pixels of Fluorescence Lifetime Images Using the Phasor Approach. *J Phys Chem B* (2020) 124(45):10126-37. Epub 2020/11/04. doi: 10.1021/acs.jpcb.0c06946.
12. Vallmitjana A, Dvornikov A, Torrado B, Jameson DM, Ranjit S, Gratton E. Resolution of 4 Components in the Same Pixel in Flim Images Using the Phasor Approach. *Methods Appl Fluoresc* (2020) 8(3):035001. doi: 10.1088/2050-6120/ab8570.
13. Ranjit S, Datta R, Dvornikov A, Gratton E. Multicomponent Analysis of Phasor Plot in a Single Pixel to Calculate Changes of Metabolic Trajectory in Biological Systems. *J Phys Chem A* (2019) 123(45):9865-73. doi: 10.1021/acs.jpca.9b07880.
14. Ranjit S, Malacrida L, Stakic M, Gratton E. Determination of the Metabolic Index Using the Fluorescence Lifetime of Free and Bound Nicotinamide Adenine Dinucleotide Using the Phasor Approach. *J Biophotonics* (2019) 12(11):e201900156. doi: 10.1002/jbio.201900156.
15. Jameson DM, Gratton E, Hall RD. The Measurement and Analysis of Heterogeneous Emissions by Multifrequency Phase and Modulation Fluorometry. *Appl Spectrosc Rev* (1984) 20(1):55-106. doi: 10.1080/05704928408081716.
16. Jameson DM. Introduction to Fluorescence. (2014).
17. Datta R, Alfonso-García A, Cinco R, Gratton E. Fluorescence Lifetime Imaging of Endogenous Biomarker of Oxidative Stress. *Sci Rep* (2015) 5:9848-. doi: 10.1038/srep09848.

2.0 SUPPLEMENTAL TABLE 1

Product Name	Manufacturer	Product Number
N-acetylcysteine	Sigma	A15409
Percoll	Cytiva	17089101
Alexa Fluor700 conjugated CD 45	BioLegend	103128
Brilliant Violet 510 conjugated CD 4	BioLegend	100553
Brilliant Violet 421 conjugated CD 8	BioLegend	100738
Brilliant Violet 605 conjugated NK-1.1	BioLegend	108740
Fluorescein isothiocyanate-conjugated CD 3	BioLegend	152304
Zombie NIR™	BioLegend	423105, 423106
Purified Rat Anti-Mouse CD16/CD32 (Fc Block™)	BD Biosciences	553142
IL-12	R&D Systems	219-IL
IL-18	R&D Systems	9124-IL
IL-15	R&D Systems	247-ILB
Cell Activation Cocktail containing phorbol-12-myristate-13-acetate	BioLegend	420601
Brilliant Violet 650™ -conjugated anti-IFN-γ	BioLegend	563854
Brilliant Violet 605™ -conjugated anti-TNF-α	BioLegend	506329
IC Fixation buffer	Invitrogen	00-5223-56 diluent, 00-5123-43 concentrate
Permeabilization buffer	Invitrogen	00-8333-56
RT² Profiler™ Polymerase Chain Reaction (PCR) Array Mouse Cytokines & Chemokines	Qiagen	330231
Allprotect Tissue Reagent	Qiagen	76405
1.0 mm Zirconia/Silica beads	BioSpec Products	11079110Z
FastPrep 24 Tissue Homogenizer	MP Biomedical	116004500
RNeasy Mini kit with RNase-free DNase set	Qiagen	74104
Xbridge Amide 3.5 µm, 4.6 × 100 mm column	Waters	SKU: 186003334
Acetonitrile	ThermoFisher Scientific	047138.M1
Water	ThermoFisher Scientific	022934.K2
NIST reference serum	NIST	909c

3.0 SUPPLEMENTAL FIGURES

A

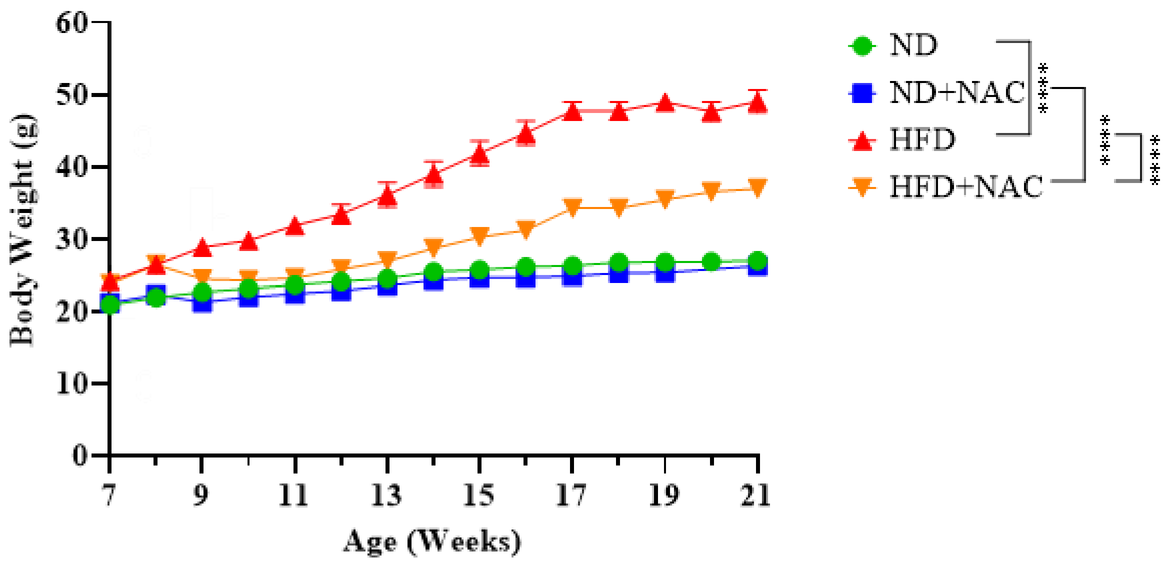

B

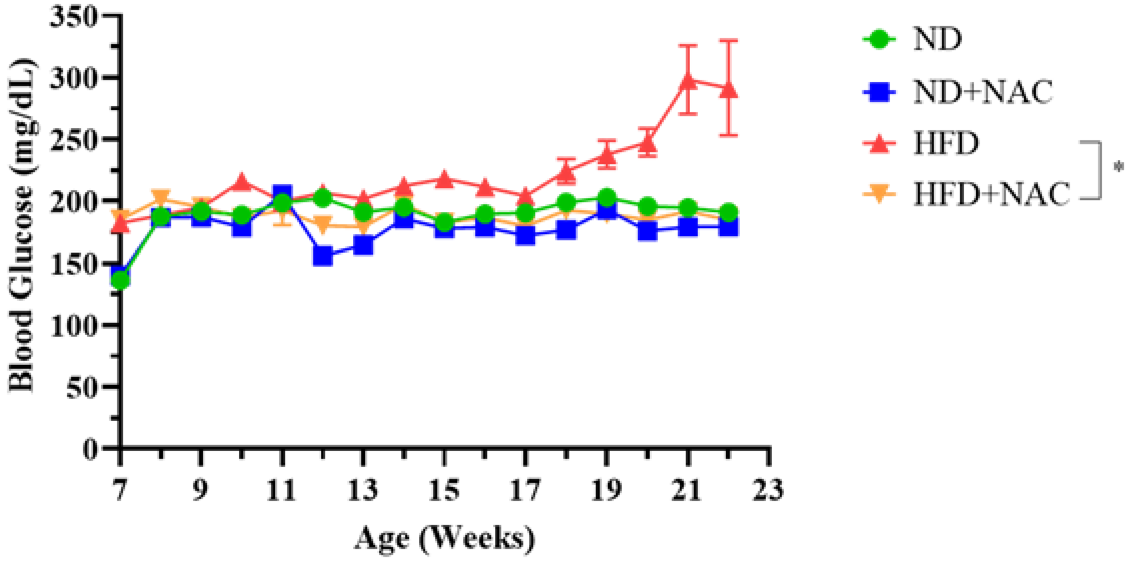

Supplemental Figure 1: Oral NAC supplementation significantly prevents weight gain in HFD mice. (A) Body weight for mice in all four groups. HFD mice gained significantly more weight than both ND and HFD + NAC mice. P-values correspond to body weights at 19 weeks of age. (B) Random blood glucose measurements. HFD mice had significantly higher blood glucose measurements starting at 12 weeks of age compared to HFD + NAC mice ($p<0.05$). P-values are annotated by "*" 0.05 to 0.01, "**"0.01 to 0.001, "***" 0.001 to 0.0001, "****" less than 0.0001

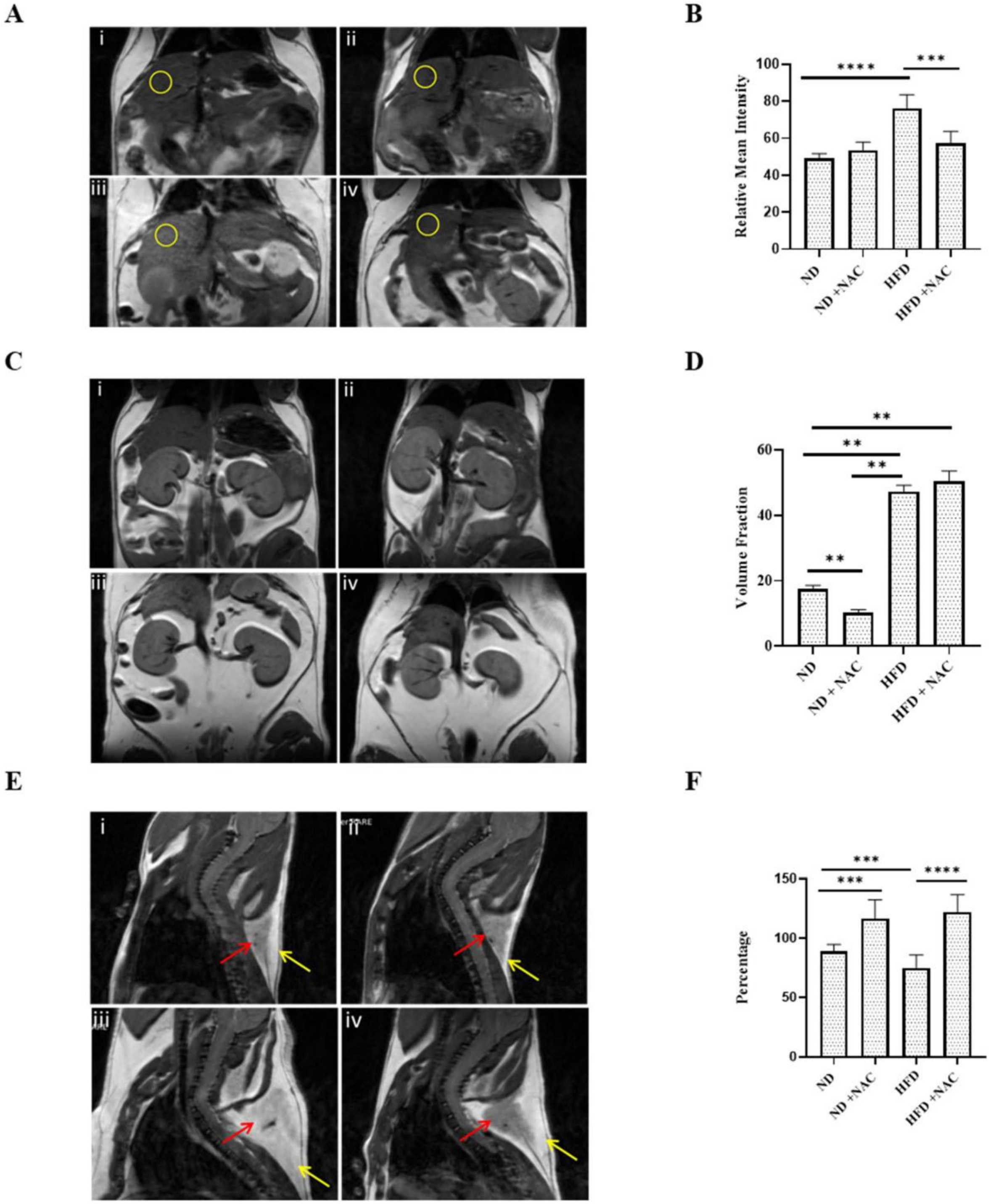

Supplemental Figure 2. T1 weighted MRI imaging of mice *in vivo*. (A-B) Hepatic Steatosis. Regions of interest (ROI) were localized on the liver of each individual animal on at least three

slices, and their mean intensity was measured and averaged to quantify hepatic fat accumulation. This demonstrated that HFD mice presented with a an ~27% increase in liver contrast, as compared to ND mice. (C-D) Abdominal fat contained in the entire dataset was quantified by thresholding with ImageJ software. Exposure to HFD caused a marked increase in abdominal fat to ~47% of the total ROI voxels, as compared with ND demonstrating ~18%. (E-F) Nuchal fat content was measured to assess metabolic and thermogenic capacity. HFD exposure resulted in the marked transformation of the BAT into WAT, as represented by the whitening of the nuchal adipose tissue.

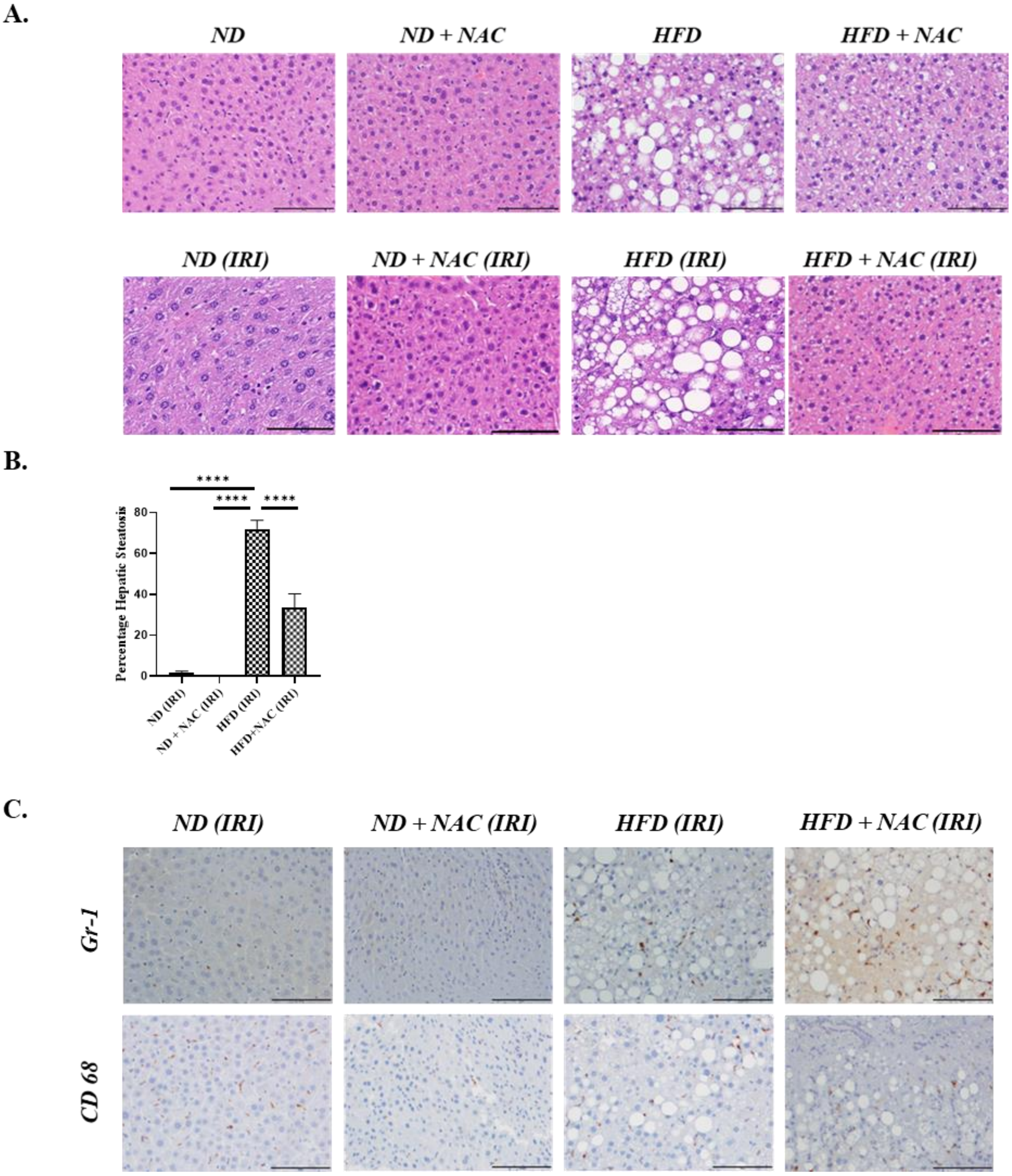

Supplemental Figure 3. H&E analysis of liver tissue. (A) H&E sections demonstrating the degree of hepatic steatosis. (B) Bar graph corresponding to the percentage of hepatic steatosis in IRI specimens. Overall, hepatic steatosis varied greatly between both ND and ND + NAC when compared to HFD mice, with HFD mice demonstrating 80% hepatic steatosis on average ($p<0.0001$). This difference was significantly reduced in HFD mice on NAC supplementation, which allowed for a 50%. (C) Representative immunohistochemical staining from mice following IRI.

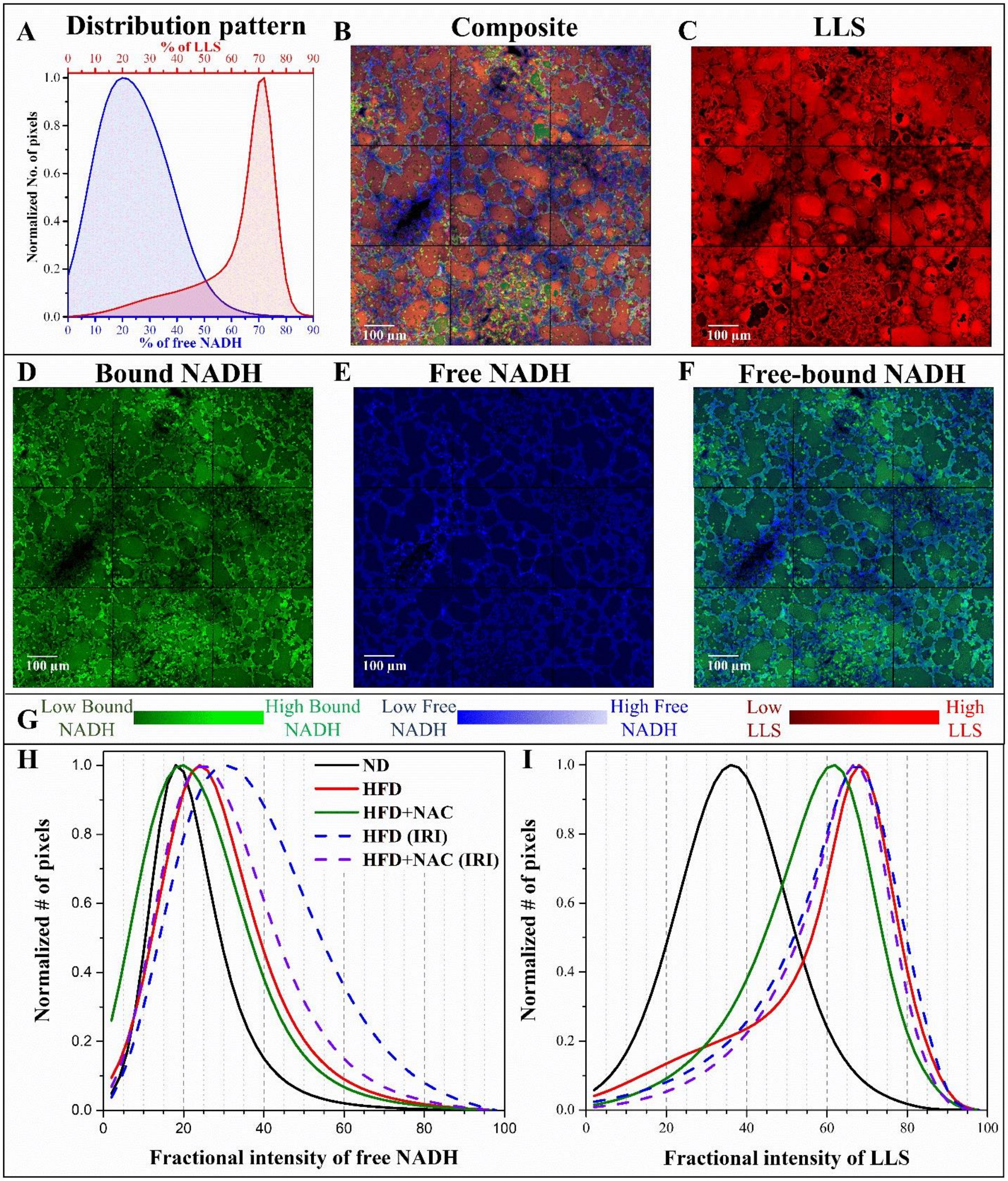

Supplemental Figure 4. Spatial Heterogeneity is an important aspect of phasor-FLIM imaging demonstrating how each sample differs in different parts of an image. The analysis represented within our work is based on quantification of components of phasor plot and their fractional intensity allows for comparison between sample to sample and between different sets of samples.

However, calculation of an average value from the total image results in loss of the dimensional information and the inherent heterogeneity that these maps can show. The actual distribution of the fractional intensity of LLS (red) and free NADH (blue) show how there is embedded spatial information if phasor-FLIM autofluorescence images (Supplemental Figure 4A. The composite image of LLS, free and bound NADH (Supplemental Figure 4B) and the individual species distribution (Supplemental Figure 4C-E) shows how different parts of the image has differential amount of these species. Supplemental Figure 4F shows the free and bound NADH map with higher blue and green representing more free and more bound NADH, respectively, and how some areas are more glycolytic than others – information that is harder to see in three color images. The cumulative graphs from 3 samples (Supplemental Figure 4 H, I) show that along with a shift of LLS and glycolysis in HFD IRI samples there could be broadening of these distributions. In case of broadening, e.g. HFD + NAC *vs* ND in Figure 4H – the central position of the distribution may not shift a lot, there is more metabolic heterogeneity in HFD + NAC samples than ND diet. Observation of the spatial difference is inherent to the imaging methods and multicomponent analysis of phasor-FLIM allows for a quantitative map of these species.

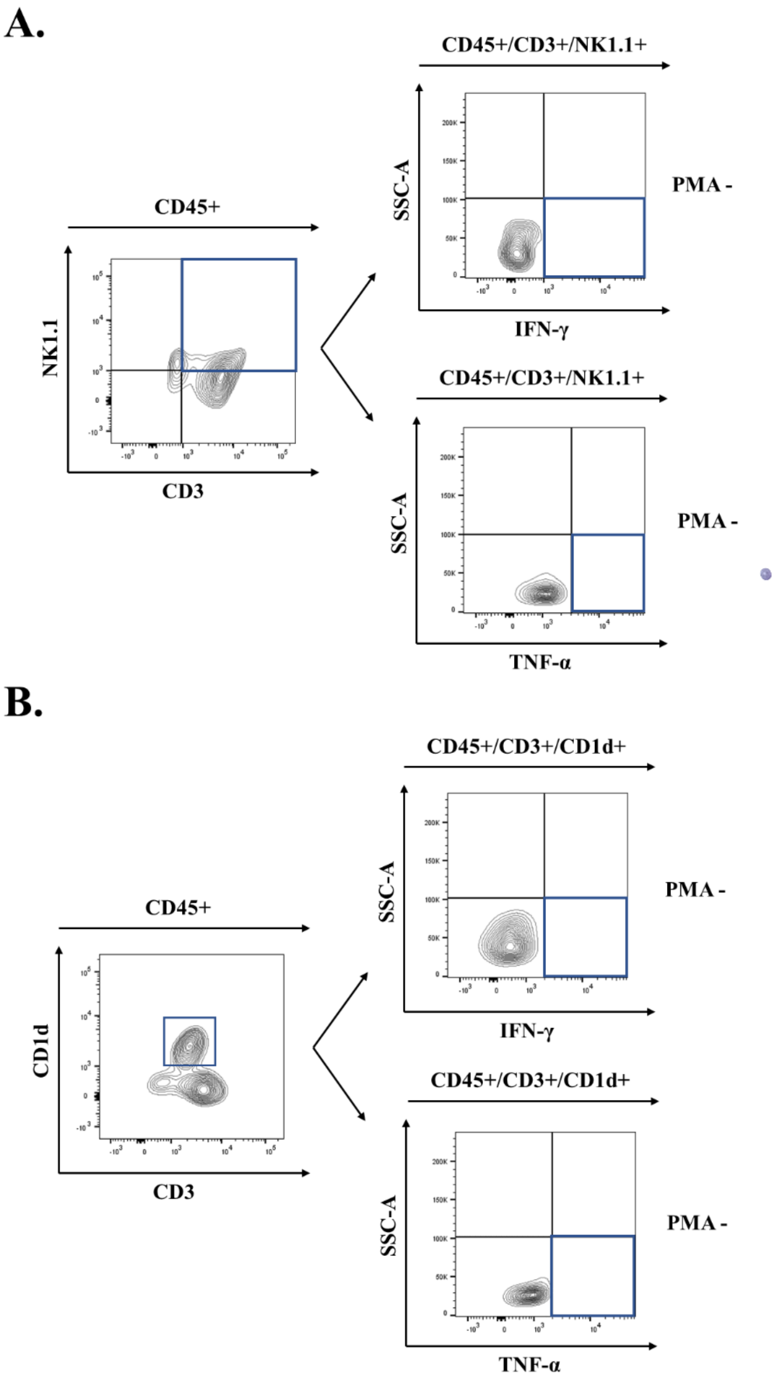

Supplemental Figure 5: Gating Strategy and stimulation negative cytokine staining controls. (A) Gating strategy for CD3+/NK1.1+ cells. All cells were gated on CD45+/Zombie- negative cell populations. IFN-γ and TNF-α cytokine staining from control tissue without PMA stimulation. (B) Gating strategy for CD3+/CD1d+ cells. All cells were gated on CD45+/Zombie-negative cell populations. IFN-γ and TNF-α cytokine staining from control tissue without PMA stimulation.

A.

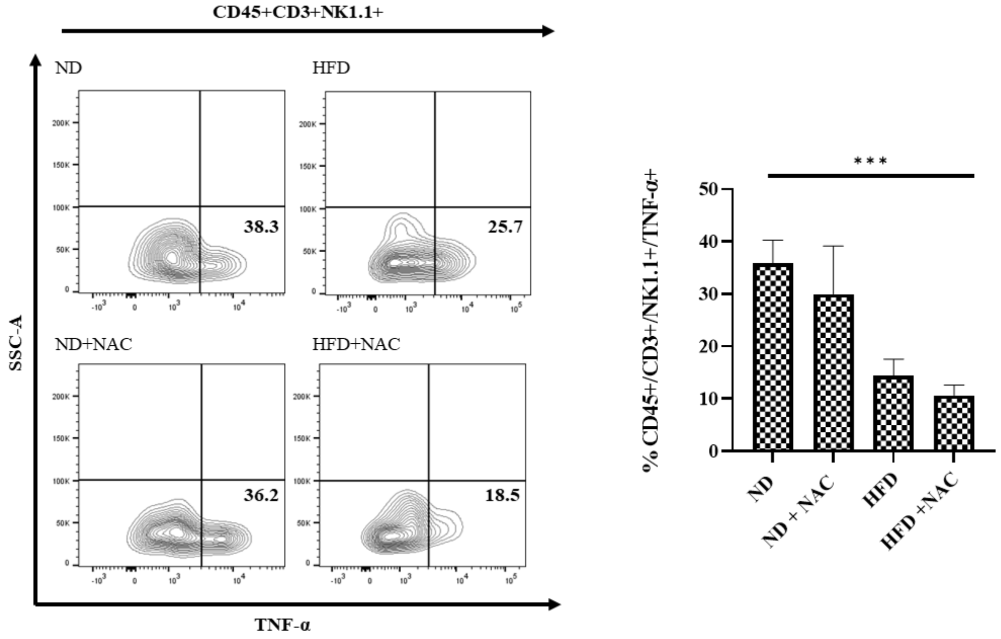

B.

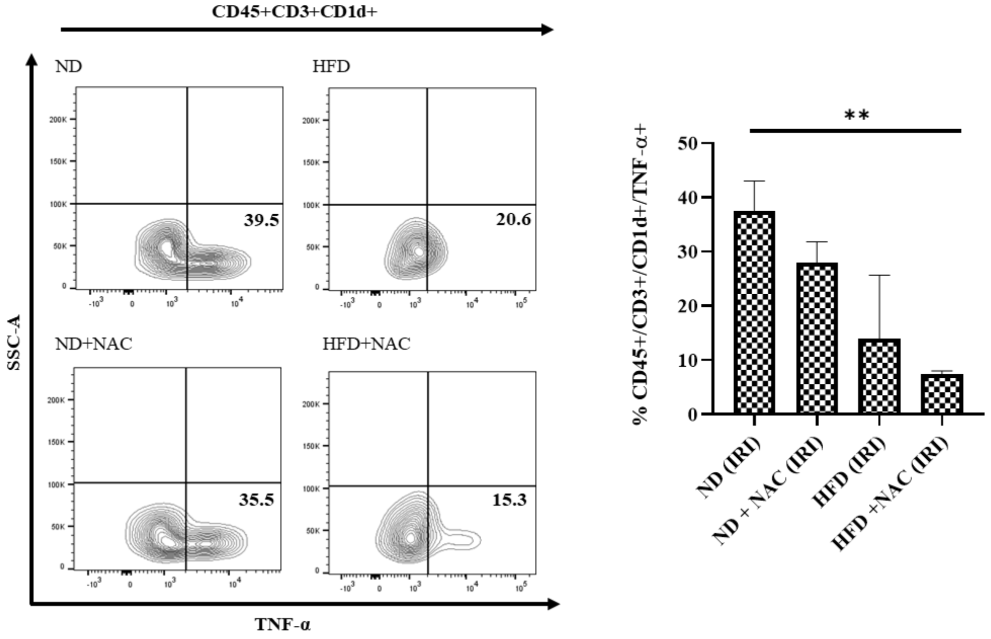

Supplemental Figure 6: Representative TNF-α cytokine staining following PMA stimulation following IRI. (A) TNF-α production from CD45+/CD3+/NK1.1+ cells with a histogram of average cytokine frequency. (B) TNF-α production from CD45+/CD3+/CD1d+ cells with histogram of average cytokine frequency.
